# G Protein Activation without a GEF in the Plant Kingdom

**DOI:** 10.1371/journal.pgen.1002756

**Published:** 2012-06-28

**Authors:** Daisuke Urano, Janice C. Jones, Hao Wang, Melissa Matthews, William Bradford, Jeffrey L. Bennetzen, Alan M. Jones

**Affiliations:** 1 Department of Biology, The University of North Carolina at Chapel Hill, Chapel Hill, North Carolina, United States of America; 2 Department of Biochemistry and Biophysics, The University of North Carolina at Chapel Hill, Chapel Hill, North Carolina, United States of America; 3 Department of Genetics, University of Georgia, Athens, Georgia, United States of America; 4 Department of Pharmacology, The University of North Carolina at Chapel Hill, Chapel Hill, North Carolina, United States of America; Fred Hutchinson Cancer Research Center, United States of America

## Abstract

Animal heterotrimeric G proteins are activated by guanine nucleotide exchange factors (GEF), typically seven transmembrane receptors that trigger GDP release and subsequent GTP binding. In contrast, the *Arabidopsis thaliana* G protein (AtGPA1) rapidly activates itself without a GEF and is instead regulated by a seven transmembrane Regulator of G protein Signaling (7TM-RGS) protein that promotes GTP hydrolysis to reset the inactive (GDP-bound) state. It is not known if this unusual activation is a major and constraining part of the evolutionary history of G signaling in eukaryotes. In particular, it is not known if this is an ancestral form or if this mechanism is maintained, and therefore constrained, within the plant kingdom. To determine if this mode of signal regulation is conserved throughout the plant kingdom, we analyzed available plant genomes for G protein signaling components, and we purified individually the plant components encoded in an informative set of plant genomes in order to determine their activation properties *in vitro*. While the subunits of the heterotrimeric G protein complex are encoded in vascular plant genomes, the 7TM-RGS genes were lost in all investigated grasses. Despite the absence of a Gα-inactivating protein in grasses, all vascular plant Gα proteins examined rapidly released GDP without a receptor and slowly hydrolyzed GTP, indicating that these Gα are self-activating. We showed further that a single amino acid substitution found naturally in grass Gα proteins reduced the Gα-RGS interaction, and this amino acid substitution occurred before the loss of the RGS gene in the grass lineage. Like grasses, non-vascular plants also appear to lack RGS proteins. However, unlike grasses, one representative non-vascular plant Gα showed rapid GTP hydrolysis, likely compensating for the loss of the RGS gene. Our findings, the loss of a regulatory gene and the retention of the “self-activating” trait, indicate the existence of divergent Gα regulatory mechanisms in the plant kingdom. In the grasses, purifying selection on the regulatory gene was lost after the physical decoupling of the RGS protein and its cognate Gα partner. More broadly these findings show extreme divergence in Gα activation and regulation that played a critical role in the evolution of G protein signaling pathways.

## Author Summary

Extracellular signals activate intracellular changes that lead to cell behaviors. This spatial coupling is mediated by cell-surface receptor activation of the heterotrimeric G protein complex located on the cytoplasmic side of the plasma membrane. Unlike the case for metazoans, plant G proteins are constitutively active. Plants use multiple mechanisms to keep the G protein complex in its resting state, and activation occurs by inhibition of this property. One mechanism involves a cell surface receptor that accelerates the return to the resting state through direct interaction with the G protein at a specific protein interface. This unique protein, AtRGS1, has both an animal like receptor domain and a domain (RGS box) responsible for accelerating deactivation. One group of plants (cereals) lost this protein through, first, a mutation in the protein interface that reduces the affinity for the RGS box to the G protein, followed by gene loss.

## Introduction

There are few well-understood examples of how signaling pathways evolved. In particular, it is not known how extant signaling molecules evolve characteristics including intrinsic activity, regulatory mechanisms and binding partners. Neutral selection theory proposes that genes released from constraints are gradually deleted from a genome. However, the processes whereby signaling genes are freed from constraints are not known and uninvestigated.

Heterotrimeric G proteins are well characterized molecular switches that are activated in response to extracellular stimuli [1], [2]. The G protein activation state is determined by the balance between rates of GDP-release (nucleotide exchange) and intrinsic GTP-hydrolysis by the Gα subunit of the heterotrimer [1], [2]. For all metazoan and yeast Gα proteins, GDP-release is slower than GTP-hydrolysis, and thus the G protein is predominantly GDP-bound in its resting state. However, both nucleotide exchange and hydrolysis are conditionally controlled by regulatory proteins in cells [3]. In animals and yeast, G protein-coupled receptors (GPCR) accelerate GDP release to favor the active GTP-bound state. Regulator of G Signaling (RGS) proteins accelerate GTP hydrolysis to favor the inactive GDP-bound state.

In contrast to this paradigm found in animals, *Arabidopsis thaliana* (Arabidopsis) Gα (AtGPA1) spontaneously self-activates without the aid of a GPCR or non-receptor GEF [4], [5]. Thus, in the absence of regulatory proteins, AtGPA1 would be predominantly GTP-bound [4], [5]. Instead, AtGPA1 inactivation is regulated *in vivo* by the single Arabidopsis RGS protein, AtRGS1 [4], [6], [7], which accelerates the intrinsically slow GTPase activity of AtGPA1 [4], [7]. AtRGS1 is the first identified protein with a domain architecture consisting of an N-terminal 7TM domain fused to an RGS domain [7], [8].

Plants and animals diverged from each other early in eukaryotic history. Based on recent evolutionary findings [9], [10], the plant kingdom is the most distinct group from animal lineages that are within Unikonts [10]. Our recent finding that G protein signaling in Arabidopsis differs greatly from that of animals raised the question of how these distinct signaling modules evolved in eukaryotes. Whether or not Bikonta other than Arabidopsis possess self-activating Gα proteins was unknown and, in cases, controversial. One group reported slow nucleotide exchange for the rice Gα [11], while another group reported relatively fast nucleotide exchange [12], albeit slower than the well-characterized Arabidopsis Gα protein [4], [5]. The Gα protein from *Glycine max* (soybean) may also possess relatively rapid GDP release [13] although there is no direct biochemical evidence supporting this idea.

Here we show that the plant kingdom employs G protein activation mechanisms distinct from those found in the animal kingdom. We analyzed plant genomes for G protein signaling components, and purified an informative subset of these components for biochemical analysis. We found that the trait of self-activating Gα was conserved throughout the plant kingdom. However, mechanisms that regulate G protein signal initiation differed throughout the plant kingdom, with some species lacking RGS proteins. We also provide evidence for the evolutionary route from one signal regulation mechanism to another. Specifically, we found in monocots that a single amino acid mutation in Gα disrupted the RGS-Gα interface and may have resulted in subsequent loss of the RGS genes from the genome. Collectively, these characteristics distinguish plant G protein signal regulation from the well-known paradigm from the animal kingdom. More broadly, this study illustrates the mechanism for how a strict functional pair (i.e. a signaling component and its regulator), commonly found in eukaryotes, was disrupted and resulted in rewiring of a cellular signaling network.

## Results

### G protein signaling components in plants

**Figure 1:**
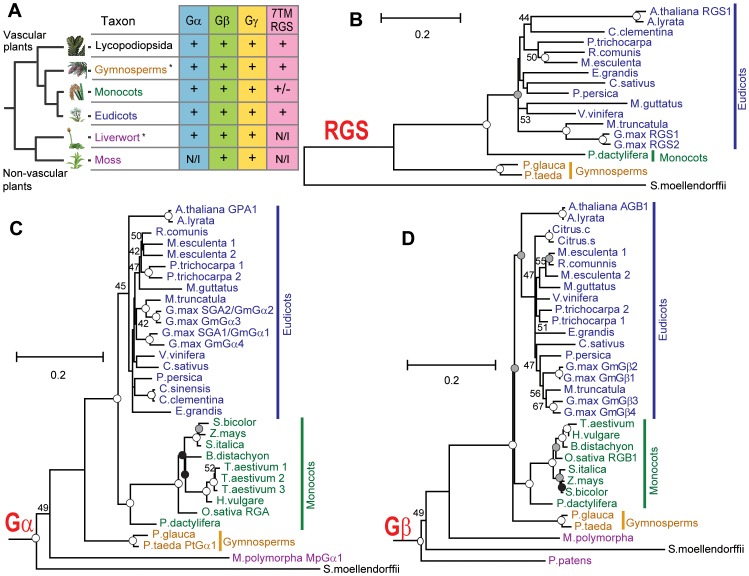
Phylogeny of G protein components in the plant kingdom. (A) Conservation of G protein components in the plant kingdom. *, Genomes have not been sequenced; N/I, Not identified; +/−, 7TM-RGS gene is identified in a date palm, but not in the grass-family. (B–D) Phylogram of the consensus maximum likelihood (ML) tree as determined for Gα, Gβ and multi transmembrane RGS protein sequences. The trees were rooted with *Homo sapiens* Gα_i1_, Gα_q_, Gβ_1_, Gβ_5_ or *S. moellendorffii* 7TM-RGS genes. Bootstrap values above 40 are shown near each branch. White, gray or black circles indicate that the branch was supported more than 90%, 80% or 70%, respectively. Species and gene names are shown in blue, green, yellow or purple colors, indicating eudicots, monocots, gymnosperms or non-vascular plants, respectively. Scale bar represents 0.1 substitutions per sites. See Figure S1, S2, S3 for aligned sequences used for creating the trees.

To identify signaling modules in the plant kingdom, homologous sequences of Gα, Gβ and Gγ genes were collected from genomic or expression sequence tags (EST) databases as described in Materials and Methods (Table 1 and Table S1, Figures S1, S2, S3). For reference, an evolutionary tree is provided in Figure 1A and includes the species described from here on. Typically vascular plants had one or two Gα genes, but *G. max*, a partially diploidized tetraploid, had four Gα genes. *Physcomitrella patens* (moss), a non-vascular land plant, lacked Gα homologs, although another non-vascular plant, *Marchantia polymorpha* (liverwort), possessed one Gα gene. One or two Gβ genes were encoded in all land plants analyzed, with the exception of soybean, which had four Gβ homologs. Likewise, Gγ genes resembling Arabidopsis Gγ genes [14], [15] were conserved in all land plants, with a few gene duplications. Notably, moss contained genes encoding the Gβγ dimer, but lacked a canonical Gα protein (Figure 1A). The moss genome encoded a gene (XP_001772174.1) homologous to Arabidopsis extra large GTP-binding protein (XLGA), although it should be noted that the moss gene lack a sequence for phosphate-binding loop (P-loop) and a glutamate residue in switch II region, each critical for G protein function.

**Table 1:** G-protein components found in genome or EST databases of the land plant kingdom.

	Gα	Gβ	Gγ	RGS^(1)^
**Eudicots**				
*Arabidopsis thaliana* *	1	1	3	1
*Arabidopsis lyrata*	1	1	3	1
*Glycine max*	4	4	8	2
*Populus trichocarpa* *	2	2	7	1
*Vitis vinifera* *	1	1	3	1
*Cucumis sativus*	1	1	6	1
*Medicago truncatula*	1	1	4	1
*Manihot esculenta*	2	2	8	1
*Ricinus comunis*	1	1	4	1
*Mimlus guttatus*	1	1	3	1
*Eucalyptus grandis*	1	1	4	1
*Citrus sinensis*	1	1	4	1
*Citrus clementina*	1	1	3	1
*Prunus persica*	1	1	3	1
**Monocots**				
*Oryza sativa* *	1	1	2	0#
*Brachypodium distachyon*	1	1	3	0#
*Sorghum bicolor* *	1	1	5	0#
*Zea mays*	1	1	5	0#
*Setaria italica*	1	1	3	1
*Triticum aestivum*	3	1	X+	0#
*Hordeum vulgare*	1	1	X+	0#
*Phoenix dactylifera* ^(2)^	1	1	X+	1
**Gymnosperms**				
*Pinus taeda* ^(3)^	1	1	3	1
*Picea glauca* ^(3)^	1	1	3	1
**Spikemoss**				
*Selaginella oellendorffii* *	1	1	1	1
**Liverwort**				
*Marchantia olymorpha* ^(3)^	1	1	1	0#
**Moss**				
*Physcomitrella patens* *	0#	1	2	0#
**Green algae**				
*Volvox carteri* *	0#	0#	0#	0#
*Chlamydomonas reinhardtii* *	0 #	0 #	0 #	0 #

Homologous genes of *A.thaliana* AtGPA1, AGB1, AGG1, AGG2, AGG3 and AtRGS1 were identified with translated protein BLAST against plant genome database through Phytozome v7.0 (released on Apr/8/2011; www.phytozome.net), PlantGDB database (http://www.plantgdb.org/), EST database registered in NCBI (www.ncbi.nlm.nih.gov), and EST database of *M. polymorpha* (More than 2 million sequence tags, http://Marchantia.pmb.lif.kyoto-u.ac.jp). (1) All RGS genes were predicted to have 7- or 5-transmembrane domain, except with non-transmembrane *S. italica* RGS. (2) DNA sequences with ∼28,000 gene predictions of *P. dactylifera* were downloaded from Weill Cornell Medical College in Qatar (http://qatar-weill.cornell.edu/research/datepalmGenome/download.html). G protein components were found with local BLAST search. (3) Sequence of G protein components was collected from EST database. Note that there might be unidentified G protein components in their species.

*: indicates species whose complete genome sequence is available from NCBI.

**+:** indicates not analyzed.

# indicates none identified.

*Chlamydomonas reinhardtii* and *Volvox carteri*, (unicellular and multicellular green algae, respectively) had no homologous genes for Gα, Gβ, Gγ and RGS, but a partial sequence of a Gα homologue was found in the EST database of *Coleochaete scutata* (a green alga, JG445935), a descendant of the most probable immediate ancestral group to land plants. These results suggest that non-vascular plant and chlorophyta lost some elements of the heterotrimeric G protein complex in their lineages.

Next, we searched for G protein regulatory elements. Previous analysis showed that plants lack canonical G protein coupled receptors [16], [17], and our analysis of new plant genomes/ESTs supported this finding. We discovered that genes encoding RGS proteins were not present in the most studied monocots, the cereals, even though RGS genes were present in all other vascular plants (eudicots, gymnosperms and a spikemoss). Although all grasses lacked a standard 7TM RGS protein, the grass, *Setaria italica* (foxtail millet) and the non-grass monocot *Phoenix dactylifera* (date palm) each possessed a gene that appears to encode an RGS protein. Unlike eudicots, however, the *S. italica* RGS lacked the transmembrane domains (Figure 1A and Table 1 and Table S1). Two eudicot RGS genes (*Ricinus comunis* and *G. max* RGS2) were predicted to have five transmembranes instead of seven transmembranes predicted for the founding member and prototype of the multi-transmembrane domain RGS family, AtRGS1. No RGS-homologous genes were found in non-vascular plants (liverwort and moss). Together, these results suggest that RGS proteins arose in an ancestor of vascular plants, but RGS genes were subsequently lost in many monocots.

We then phylogenetically analyzed the evolution of G protein signaling components. Generally, phylogenies of genes encoding plant Gα, Gβ and 7TM-RGS matched those generated with other genes used for phylogeny construction [18] (Figure 1*B*–1*D*). Near the end of angiosperm evolution, monocot and eudicot Gβ had approximately the same branch length from the common ancestor (Figure 1*D*). However, Gα evolution was accelerated in the monocot branch: the branch length of Poaceae (grass family) Gα from the common ancestor with *P. dactylifera* was almost twice as long as that of date palm Gα (Figure 1*C*). We hypothesize that this accelerated evolution of monocot Gα subunits compensated for the loss of RGS genes and/or was the result of the loss (discussed below).

### The “self-activating” trait of the Gα protein is conserved in the plant kingdom

**Figure 2:**
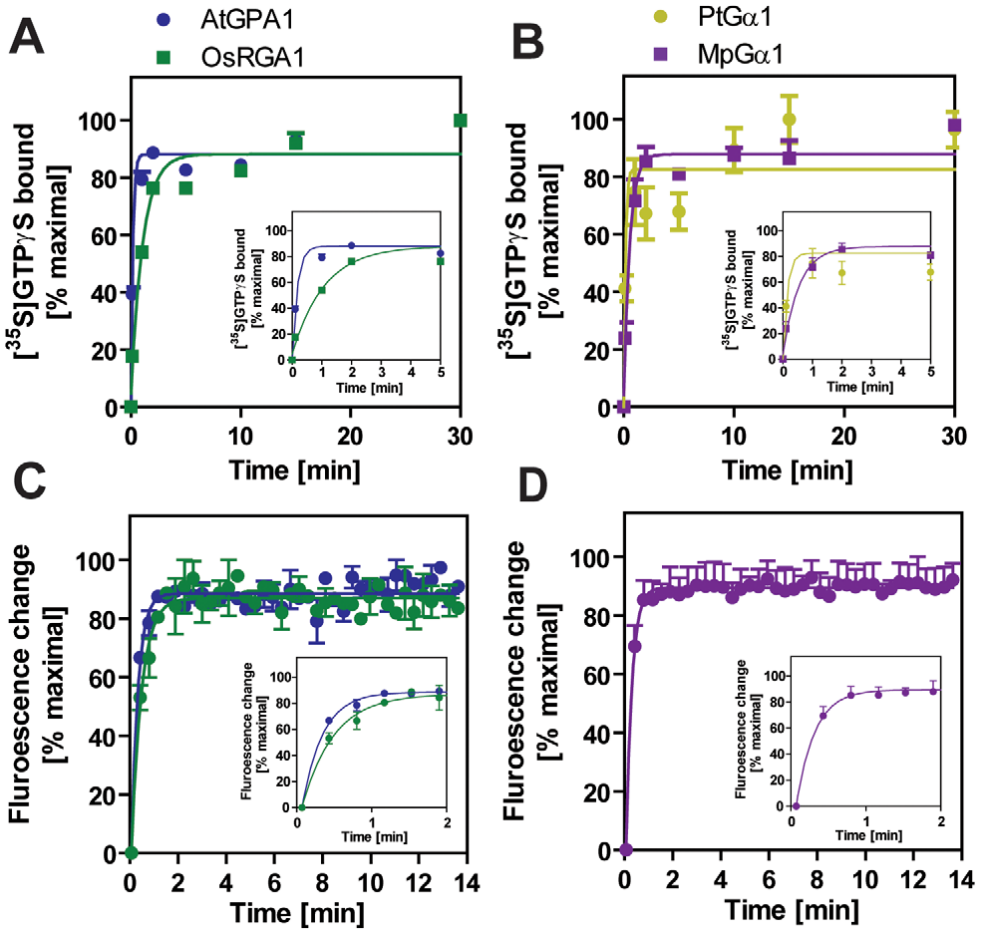
Activation of plant Gα subunits. (A, B) Time course of [^35^S]GTPγS binding to 1 µM Gα at 20°C. Data are presented as the mean ± SEM of more than triplicates. (C, D) Intrinsic tryptophan fluorescence of Gα was measured at room temperature. 5 µM GTPγS was added to 400 nM Gα in the cuvette at time 0. Data are mean ± SEM of duplicate samples.

We included representatives from a eudicot (*A. thaliana* AtGPA1), a grass *(Oryza sativa* OsRGA1), a gymnosperm *(Pinus taeda* PtGα1), and a nonvascular plant (*M. polymorpha* MpGα1). First, we characterized the nucleotide exchange rates of these proteins using the non-hydrolysable GTP analog, GTPγS (Figure 2B and Table 2). Consistent with previous results [4], [5], we found that AtGPA1 had fast nucleotide exchange (K_obs_ = 5.80 min^−1^). In contrast to an early report that suggested OsRGA1 had slow nucleotide exchange [12], we found that OsRGA1 exchange nearly matched that of AtGPA1 (OsRGA1, K_obs_ = 0.92 min^−1^). Our value is similar to that published in other studies [4], [5], [12]. Likewise, Gα from pine (PtGα1, K_obs_ = 6.85 min^−1^) and liverwort (MpGα1, K_obs_ = 1.84 min^−1^) also had fast nucleotide exchange. These nucleotide exchange rates were corroborated by measuring the rate of the activation-dependent change in intrinsic Gα fluorescence [19], [20] (Figure 2*C and 2D*). Together these data suggest that the trait of fast GDP release is conserved in land plants, and likely arose in a common ancestor of this super group (Table 2).

**Table 2:** Rates of nucleotide exchange and GTP hydrolysis.

	[35S]GTPγS Binding	GTPγS binding (Fluoresence)	[γ-^32^P]GTP hydrolysis	Rate-limiting step	% GTP bound
AtGPA1	5.80±0.93	3.63±0.38	0.047±0.007	Hydrolysis	99
AtGPA1 T194N	6.97±1.02	N/A	0.059±0.004	Hydrolysis	99
OsRGA1	0.92±0.12	2.38±0.24	0.052±0.004	Hydrolysis	95
OsRGA1 N195T	0.70±0.06	X+	0.051±0.006	Hydrolysis	93
PtGα1	6.85±2.21	0*	X+	X+	X+
MpGα 1	1.84±0.36	4.09±0.61	0.87±0.19	Hydrolysis	68

Nucleotide exchange was measured with intrinsic Trp fluorescence and with [^35^S]GTPγS binding. Rate of GTP hydrolysis was measured with single turn [γ-^32^P]GTP hydrolysis. The rate-limiting step was determined by comparing the rates of GTP binding and hydrolysis. The percentage of bound GTP was approximated by the following equation: GTP binding rate/(GTP binding rate + GTP hydrolysis rate). Note that GTP/GDP ratio and Gα interacting proteins will affect the state in vivo. Rates were measured at 20°C and are reported as min^−1^.

*: Fluorescence change was not detected, likely because of low specific activity of the recombinant protein.

**+:** not analyzed.

**Figure 3:**
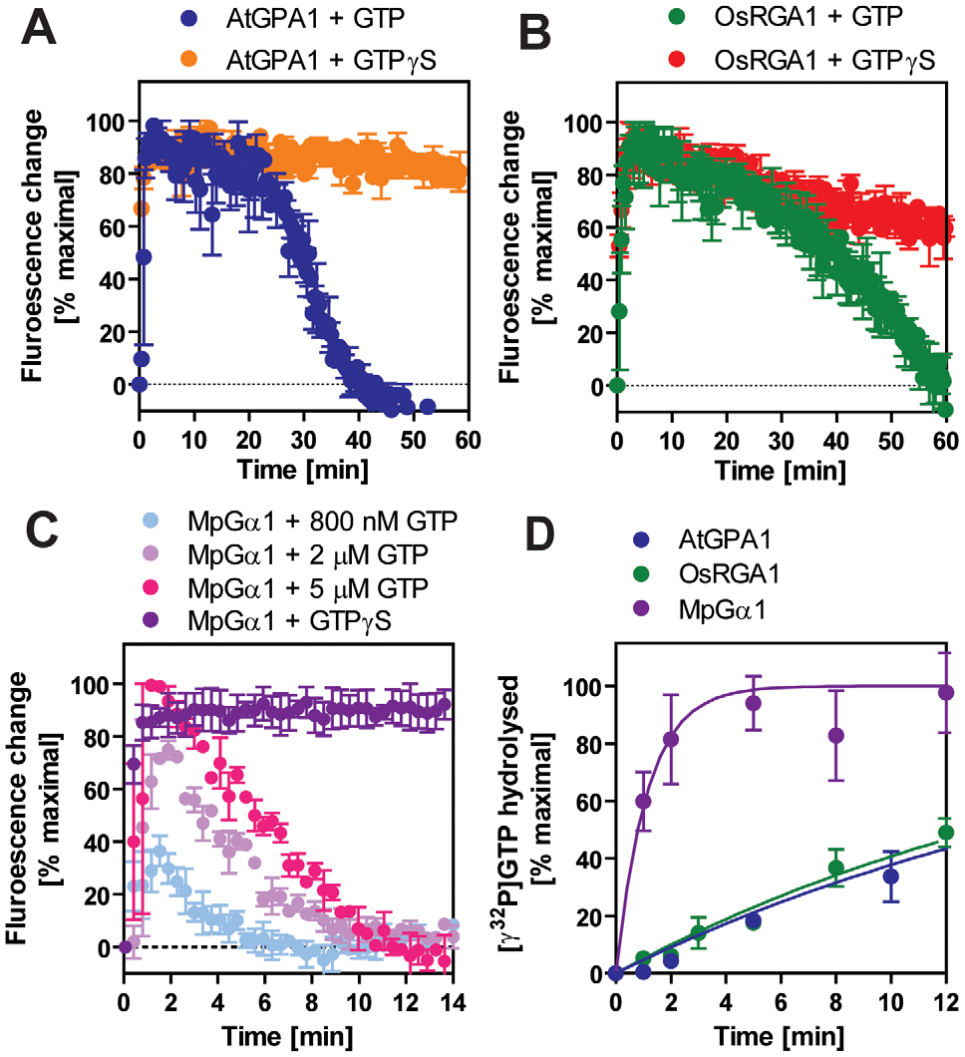
Inactivation of plant Gα subunits. (A–C) The activation and inactivation rates of Gα were monitored at room temperature by measuring the intrinsic tryptophan fluorescence. 800 nM GTP or 5 µM GTPγS was added to 400 nM Gα in the cuvette at time 0. Data are mean ± SEM of duplicate samples. Note that the turnover rate of Gα depends on stoichimometry of the concentrations of active Gα protein and GTP. The specific activity of the Gα subunits estimated by [^35^S]GTPγS binding assay were as follows: AtGPA1, 0.46 mol/mol protein; RGA1, 0.55 mol/mol protein; MpGPA1, 0.69 mol/mol protein. (D) Time course of single turnover [γ^32^P]GTP hydrolysis by 800 nM Gα. The mean ± SEM of duplicate samples is shown.

For a Gα protein to be called “self-activating,” it must release GDP/bind GTP faster than it hydrolyzes GTP. In other words, the Gα should accumulate in its active form without a regulatory protein. Thus, we measured the rate of Gα-GTP accumulation in the presence of hydrolysable GTP (Figure 3*A*–3*C*). In this reaction, activated Gα would only be observed if the rate of nucleotide exchange was faster than the rate of GTP hydrolysis (i.e. when the Gα protein is “self-activating”) [5]. All tested plant Gα subunits accumulated in the active state with GTP (Figure 3A–C). Grass Gα (OsRGA1) and eudicot Gα (AtGPA1) displayed sustained activation in the presence of GTP. However, the Gα from liverwort (MpGα1) quickly returned to the inactive form, even in the presence of a 10-fold molar excess of GTP (Figure 3C), suggesting that the liverwort Gα had a fast rate of GTP hydrolysis. To test this hypothesis, we directly measured the intrinsic rates of inactivation of the selected Gα subunits by quantifying release of ^32^PO_4_ from [γ-^32^P]GTP in single turnover GTPase experiments (Figure 3*D* and Table 2). AtGPA1 (K_cat_ = 0.047 min^−1^ [4], [5]) and OsRGA1 (K_cat_ = 0.052 min^−1^) had slow rates of GTP hydrolysis. In contrast, liverwort MpGα1 (K_cat_ = 0.87 min^−1^) had a 16-times faster GTP hydrolysis rate than AtGPA1 and OsRGA1, indicating that MpGα1 efficiently inactivates itself without an RGS protein, yet hydrolysis remains the rate-limiting step. Together, these results suggest that land plant Gα subunits are all “self-activating” due to rapid nucleotide exchange relative to GTP hydrolysis and that the controlled step for activating G signaling is at GTP hydrolysis. The ideal element for this control is a 7TM-RGS protein, represented by the prototype AtRGS1. However, the absence of 7TM-RGS proteins in grasses indicates an alternative regulatory mechanism must exist in this class.

### Grass-specific loss of the 7TM-RGS gene was preceded by a single amino acid mutation on Gα

**Figure 4:**
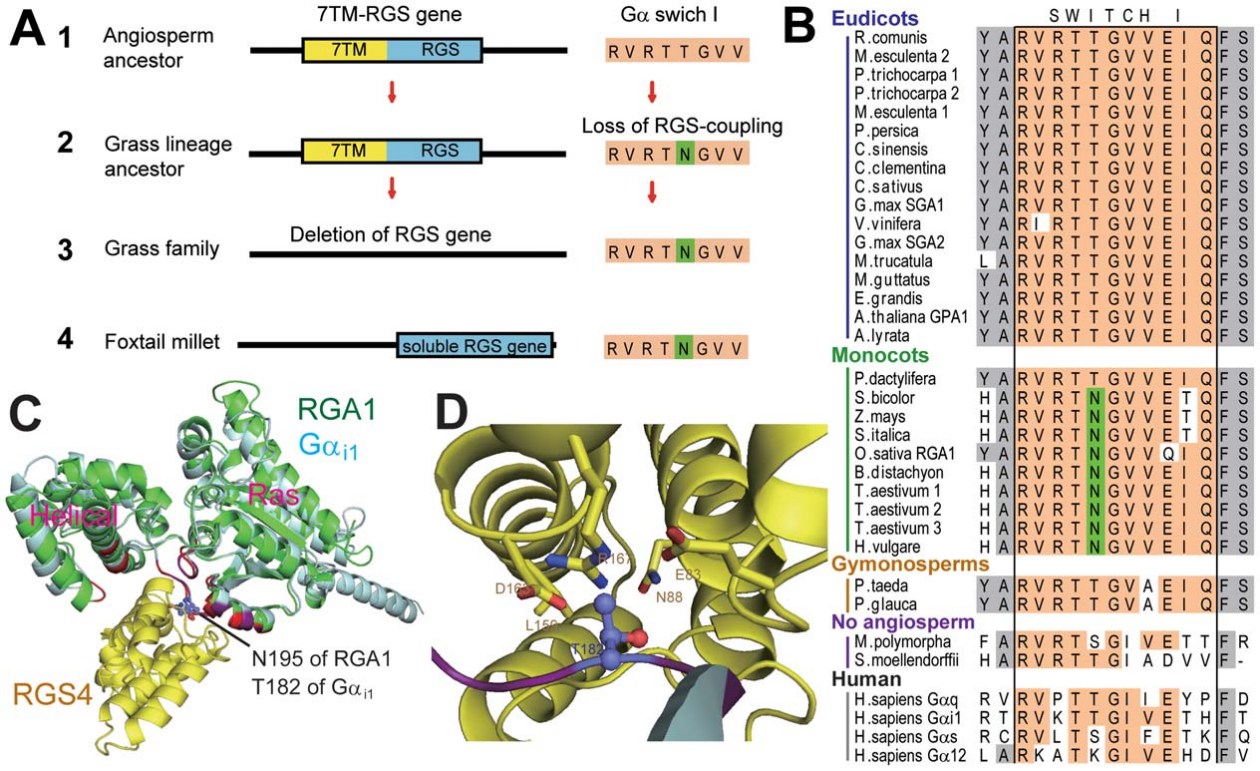
Evolution of monocot Gα and monocot specific loss of a 7TM-RGS gene. (A) Evolutionary process to delete a 7TM-RGS gene in monocots. 1. The angiosperm ancestor possessed a 7TM-RGS gene. 2. After separation from the palm tree lineage, the Gα in the grass lineage lost a functional and physical coupling with its partner RGS protein. This uncoupling occurred by a single amino acid mutation, thus releasing the RGS gene from evolutionary constraint. 3. RGS genes were gradually deleted from grass-family genomes. 4. The extant *S.italica* RGS gene may still be functional, despite the loss of the 7TM region. (B) Switch I region of Gα. Conserved residues are highlighted with orange. Substituted Asn residue in monocots (Asn195 of OsRGA1) is shown with green. See Table S1 for species names. (C) Predicted structures of OsRGA1 (green) overlayed on *Rattus norvegicus* Gα_i1_ and RGS4 (cyan and yellow, PDB: 1AGR [21]). Residues in purple (Gα_i1_) or red (OsRGA1) are at the binding surface to RGS4. Asn195 of OsRGA1 and Thr182 of Gα_i1_, discussed in this paper, are illustrated by a stick model. (D) The binding surface between Gα_i1_ and RGS4. Thr182 of Gα_i1_ and the interacting residues of RGS4 are shown in a stick model.

Under neutral selection, genes freed from evolutionary constraint are rapidly deleted from the genome. This implies that 7TM-RGS was released from the strict functional constraint with Gα early in grass family history. To determine how this release may have occurred, we modeled the putative RGS - grass Gα protein interaction interface (Figure 4*C and 4D*) and found that a threonine residue in switch I (Thr194 of AtGPA1) that is critical for interaction with RGS proteins [21] was changed to asparagine in most monocot Gα subunits (Asn195 of OsRGA1, Figure 4*B*). This threonine residue is conserved in the RGS-sensitive human Gα_i_ and Gα_q_ family (Figure 4*B*) and is located at the center of the interface with RGS protein [21]. The threonine residue is substituted to lysine in Gα_12_ and Gα_13_, and this class of Gα subunits has dedicated RGS Homology (RH) proteins of Rho-family GEFs. Gα_12_ and Gα_13_ are not substrates for RGS proteins, which are dedicated GAPs of Gα_i_ and Gα_q_. A mutation of this lysine of human Gα_13_ abolishes interaction with the RGS domain of a Gα_13_ effector, leukemia-associated Rho GEF (LARG) [22].

Two monocots were atypical in that they retained RGS-encoding genes. Examination of Gα and RGS sequences from these monocots provided insight into how other monocots may have lost RGS genes. First, the RGS protein in the monocot *P. dactylifera* has the typical (i.e. the Arabidopsis prototype) 7TM-RGS topology and its gene transcription is supported by EST data (Figure S4). The *P. dactylifera* Gα has the threonine critical for RGS interaction (Figure 4*A*). Notably, the *P. dactylifera* gene is longer than the Arabidopsis AtRGS1 gene by 19 kb, primarily due to the dramatic expansion of intron between the 7TM and RGS domains (Figure S4A) suggesting that this part of the gene was subjected to DNA insertion, possibly through transposon activity. The monocot *S. italica* also encodes a single soluble RGS gene. However, the *S. italica* Gα has Asn instead of Thr in the switch I region (Figure 4*A*). These analyses indicate that grass Gα subunits lost the ability to couple with RGS, thus releasing the genetic linkage between the Gα and the RGS protein, although it is also possible that deletion of RGS genes in grasses preceded the Gα mutation.

To trace the evolutionary process leading to the deletion of the 7TM-RGS gene in grasses, we surveyed *S. italica* genomic sequences surrounding the coding region of the single RGS gene (SiPROV019851m), and we found a hypothetical gene upstream of the RGS gene (SiPROV032159m) with sequence homologous (E value = 1e^−28^) to transmembrane helices 2 through 4 of AtRGS1 (Figure S5 and Table S2). We also found nine ESTs that were homologous to the RGS domain. However, we found no sequence homologous to the region upstream of the 7TM domain (Figure S6). Moreover, the ratio of change in synonomous vs. nonsynonous residues (dn/ds) in comparing the *S. italica* homologies to their orthologs in date palm and Arabidopsis were higher for the 7TM region than for the RGS region. This suggests that the *S. italica* RGS domain continued with a function that was under strong purifying selection while the *S. italica* 7TM domain, although under purifying selection for most of the last 100 million years, has been under neutral or diversifying selection for the last few thousands or millions of years (Table S3).

Closer examination of the assembled sequence provided a clue to the partial gene loss. In the *Setaria* RGS region, we found two transposons inserted between the conserved and transcribed RGS domain and the apparent 7TM domain (Figure S6). Lack of EST support, suggest that the 7TM domain became a pseudo gene. One insertion is a partial sequence of a LINE transposon, likely resulting from deletion after insertion because the polyA and target site duplication (TSD) are missing. The second insertion was of a previously unknown long terminal repeat (LTR) retrotransposon that we named Alubu. Unequal homologous recombination subsequently converted this insertion into a solo LTR with intact TSD. There are 2 additional Alubu solo LTRs (but no complete elements) in the current *Setaria* sequence assembly (phytozome 7 http://www.phytozome.net/).

To identify other possible remnants of the 7TM-RGS gene in other grasses that lack RGS genes, we performed a tBLASTx search using the genomic sequences of the *S. italica* RGS-homologous region (segment 13, bases 1356001–1363646) against other monocot genomes. No homologous sequence of *S. italica* RGS was found in the other grasses analyzed, although the possibility of highly divergent RGS genes in plant genomes is not excluded.

These results indicate that a vascular plant ancestor had the 7TM-RGS gene. Furthermore, these analyses suggest that grasses gradually lost the RGS gene once it was uncoupled from the Gα protein by mutation of the RGS-Gα interaction interface. More broadly, these analyses point to the mechanism whereby a single amino acid substitution can lead to rewiring of a new signaling network In this case, the mutation led to neutral selection and loss of a regulatory element from the signaling pathway.

### Physical uncoupling between RGS and monocot Gα

**Figure 5:**
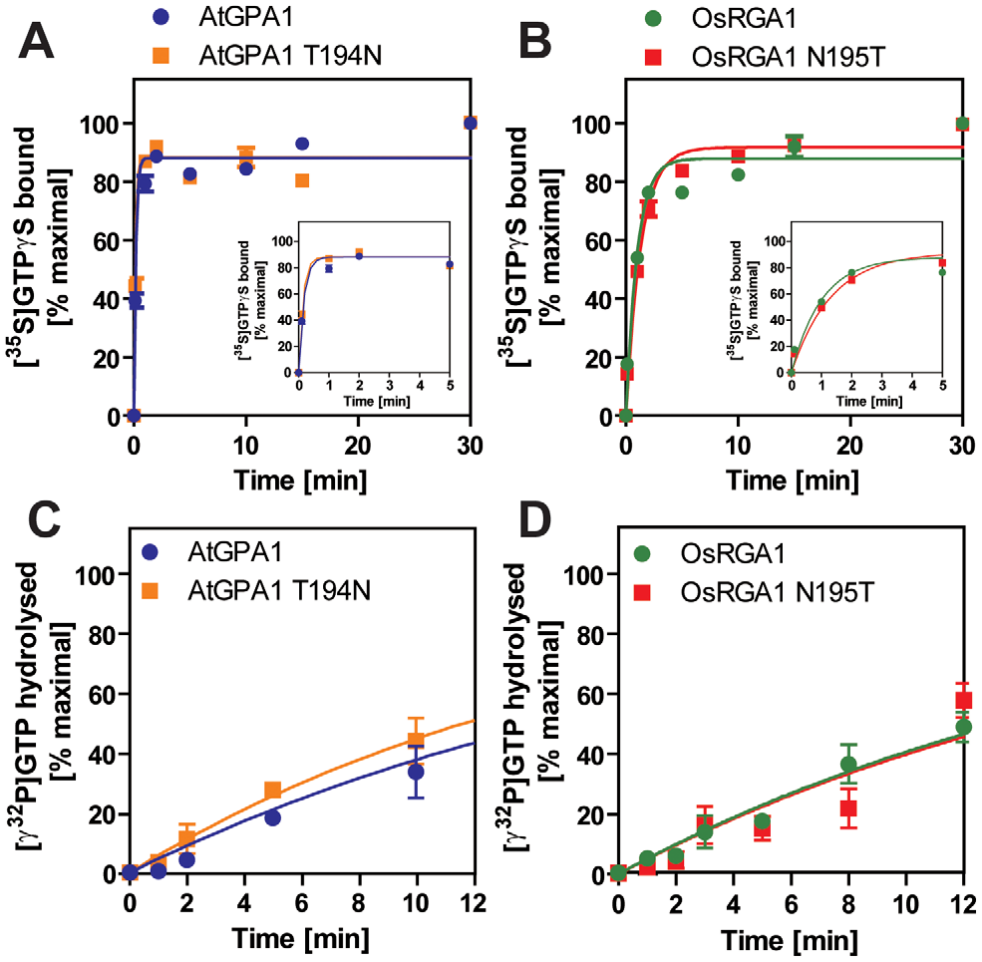
Intrinsic properties of Gα mutants. (A, B) Time course of [^35^S]GTPγS binding to 1 µM Gα at 20°C. Data are presented as the mean ± SEM of triplicates. (C, D) Time course of single turnover [γ^32^P]GTP hydrolysis by 800 nM Gα. The mean ± SEM of duplicate samples is shown.

**Figure 6:**
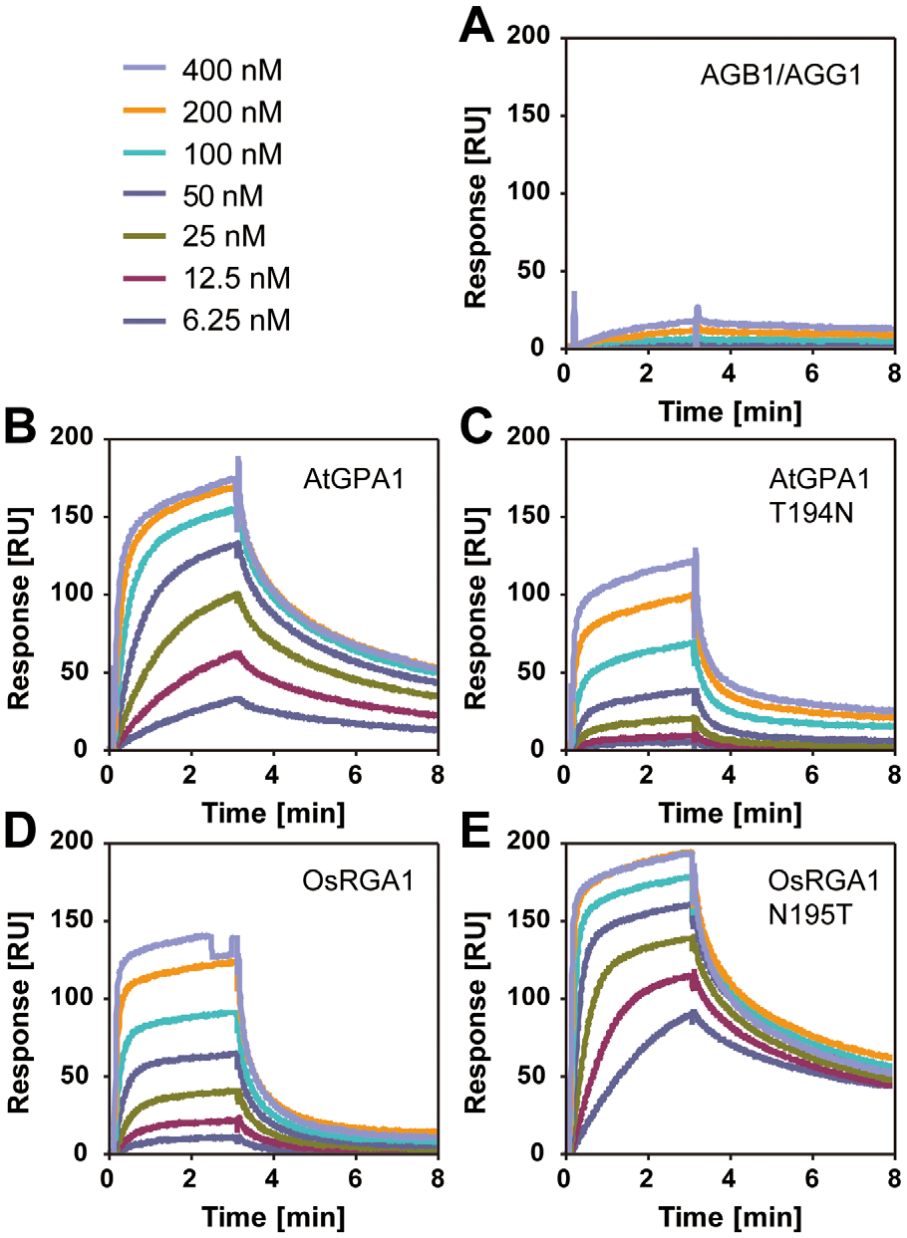
Affinity of plant Gα to AtRGS1 immobilized on the SPR biosensor. Recombinant AtRGS1 (284–459 aa) was immobilized on sensor chip CM5. AlF_4_-bound Gα subunits or the Gβγ dimer control (A) flowed over the chip at seven different concentrations (6.25, 12.5, 25, 50, 100, 200, 400 nM). Kinetics determined with 1∶1 (Langmular) binding model is shown in Table 3. Wild type Arabidopsis Ga subunit (B), T194N mutant Arabidopsis Gα subunit (C), wild type rice Gα subunit (D), and 195T mutant rice Gα subunit (E).

**Figure 7:**
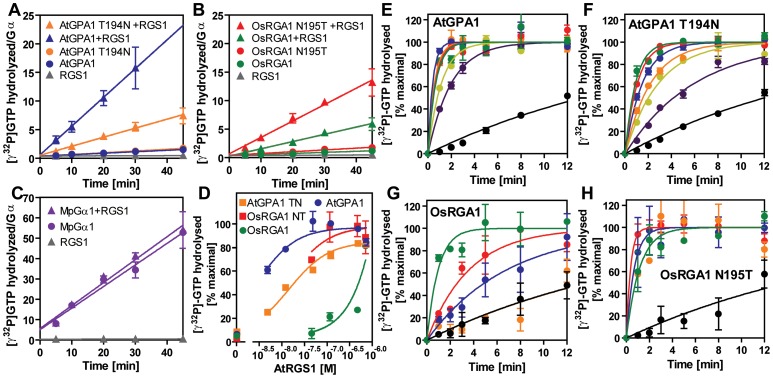
GAP activity of RGS toward plant Gα subunits. (A–C) Time course of steady-state [γ^32^P]GTP hydrolysis by 500 nM Gα in the presence or absence of 750 nM AtRGS1 were measured over time after incubation at 20°C. Rate of Pi production (mol/mol Gα protein) were shown. Data are mean ± SEM of duplicate samples. (D–H) Single turnover [γ^32^P]GTP hydrolysis by 500 nM Gα proteins with AtRGS1 (0 nM (black), 5 nM (purple), 12.5 nM (yellow), 50 nM (orange), 125 nM (blue), 500 nM (red) and 750 nM (green)). Data are mean +/− SEM for more than two individual experiments, except OsRGA1 with 50 nM RGS at 1 min and 5 min. Dose dependency of single time point (2 min) was shown in (D).

Our bioinformatics analyses suggested that the single amino acid substitution in the Gα protein-RGS interface was sufficient to release the RGS protein from evolutionary linkage to the Gα protein. To test this hypothesis experimentally, we substituted the threonine with an asparagine in the extant Arabidopsis Gα protein (AtGPA1-T194N) to recapitulate the monocot RGS interaction interface. We also made the comparable reverse substitution in OsRGA1, a representative monocot Gα protein. These mutations did not affect intrinsic nucleotide exchange and GTP hydrolysis rates (Figure 5*A*–5*D*). Next, we quantified interaction between these Gα proteins and the RGS protein from Arabidopsis (Figure 6*A*–6*E* and Table 3). As shown by surface plasmon resonance (SPR) analysis, AtGPA1 had high affinity for AtRGS1 (K_D_ = 17.4 nM), and OsRGA1 had a relatively lower affinity for AtRGS1 (K_D_ = 56.7 nM). We next tested two mutated Gα subunits, AtGPA1-T194N and OsRGA1-N195T. Although these mutations did not affect intrinsic nucleotide exchange and GTP hydrolysis rates (Figure 5*A*–5*D*), the T194N mutation reduced AtGPA1 affinity for AtRGS1 by 7-fold (K_D_ = 115 nM). Reciprocally, the N191T mutation increased OsRGA1 affinity for AtRGS1 by 12-fold (K_D_ = 4.83 nM). As a second measure of RGS-Gα interaction, we quantified GTPase acceleration by AtRGS1 in a steady-state GTP hydrolysis experiment (Figure 7*A*–7*C* and Table 4). Consistent with the affinities from SPR analysis, the T194N mutation of AtGPA1 reduced the GTPase acceleration by AtRGS1, and the N195T mutation of OsRGA1 increased GTPase acceleration by AtRGS1. This change in RGS1 sensitivity conferred by single amino acid substitution was confirmed using enzyme titration assays (Figure 7*D*–7*H*). Notably, intrinsic GTP hydrolysis by liverwort MpGα1 was fast (1.1±0.1 min^−1^) and was not further stimulated by AtRGS1. These results suggest evolution of distinct regulatory systems of plant G proteins in the eudicots, grasses and liverworts.

**Table 3:** Affinity of plant Gα to AtRGS1 immobilized on the SPR biosensor.

	k_a_ [M^−1^ s^−1^]	k_d_ [s^−1^]	K_D_ [M]
AtGPA1	3.53×10^5^	6.16×10^−3^	1.74×10^−8^
AtGPA1 T194N	1.17×10^5^	1.34×10^−2^	1.15×10^−7^
OsRGA1	3.83×10^5^	2.17×10^−2^	5.67×10^−8^
OsRGA1 N195T	1.21×10^6^	5.84×10^−3^	4.83×10^−9^

Recombinant AtRGS1 (284–459 aa) was immobilized on sensor chip CM5. Active form of Gα subunits was injected at seven different concentrations (6.25, 12.5, 25, 50, 100, 200, 400 nM). Kinetics shown in table was determined with 1∶1 (Langmular) binding model. k_a_, association rate constant; k_d_, dissociation rate constant; K_D_ = k_d_/k_a_, equilibrium constant.

**Table 4:** Rates of steady state GTP hydrolysis.

	Minus RGS	Plus RGS
AtGPA1	0.025±0.005	0.503±0.086
AtGPA1 T194N	0.031±0.005	0.158±0.019
OsRGA1	0.019±0.004	0.127±0.016
OsRGA1 N195T	0.031±0.006	0.288±0.029
MpGα1	1.06±0.08	1.13±0.12

Steady state GTP hydrolysis [min^−1^]. Steady-state [γ-^32^P]GTP hydrolysis was measured as described in Figure 7 at 20°C and reported as min^−1^.

Collectively, our phylogenetic and biochemical analyses suggest that the grass Gα lost the ability to interact with the regulatory molecule early in the evolutional lineage by substituting one critical residue (Figure 4*A*). The substitution of threonine to asparagine likely occurred in grasses before the loss of the RGS protein, a stage represented in *S. italica*, which contains the asparagine substitution, yet still encodes a remnant trace of the 7TM-RGS gene. This suggests that the physical uncoupling of Gα with RGS by single amino acid mutation broke the signaling pathway linkage permitting the subsequent deletion of RGS genes in grasses (Figure 1B).

## Discussion

### Evolution of “self-activating” Gα and 7TM-RGS

**Figure 8:**
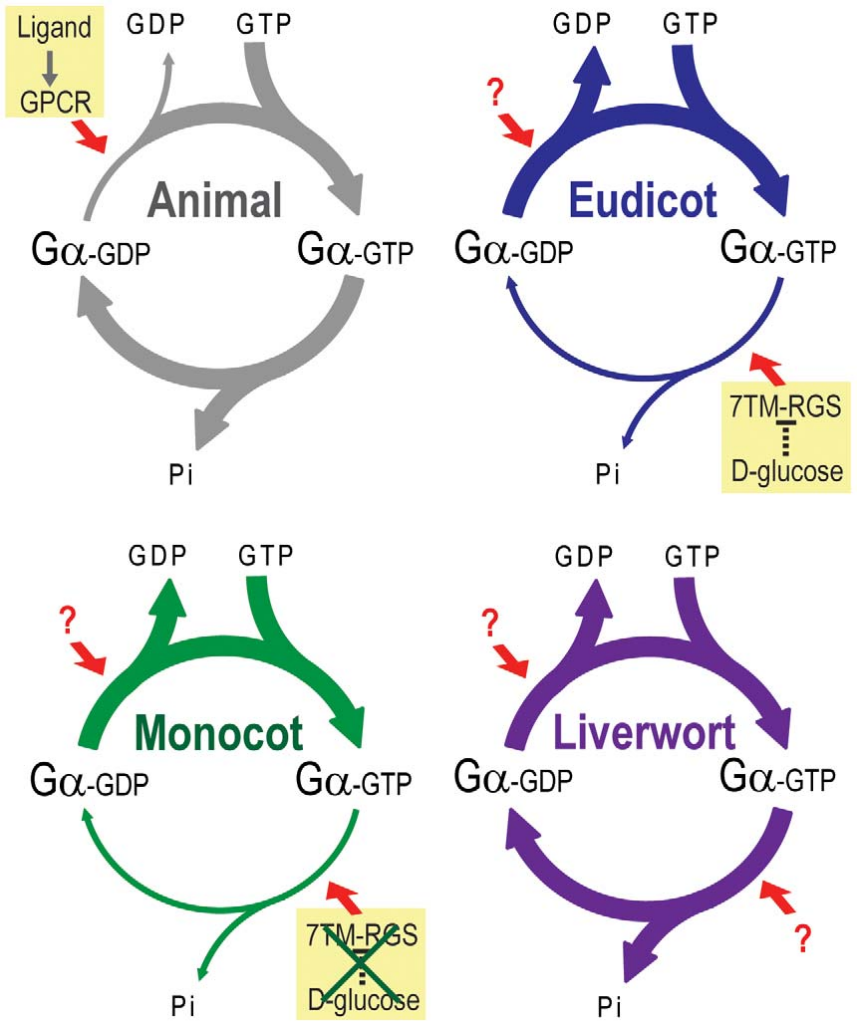
Model of G protein activation in the plant kingdom. Slow rate of GDP release and GTP hydrolysis is indicated by a thin arrow. A rapid rate is indicated by a thick arrow. In animals, a rate of GDP release from Gα is much slower than that of GTP hydrolysis. Thus, acceleration of the GDP release by GPCR changes the G protein from inactive to active. In eudicots and monocots, GDP release is rapid, and GTP hydrolysis is much slower than the GDP release. Thus, G protein can self-activate without the aid of a GPCR or other GEF. Instead, the eudicot G protein is regulated by a 7TM-RGS protein, which constitutively promotes GTP hydrolysis step on plasma membrane. However, some monocot genomes lack the 7TM-RGS gene, thus some monocot G protein must use an unknown mechanism to regulate activation. In addition, a 7TM-RGS gene is not expressed in a liverwort. However, a liverwort G protein has a rapid rate of both GDP release and GTP hydrolysis, which is likely to compensate for the loss of the 7TM-RGS gene.

GDP release and GTP hydrolysis by Gα proteins are balanced to establish the steady-state level of the activated Gα subunit of the heterotrimeric G protein complex. In animals, G protein coupled receptors alter this balance to favor the GTP-bound state and relay signals from the outside of the cell to the inside of the cell. Likewise, RGS proteins accelerate GTP hydrolysis to favor the GDP-bound state and terminate intracellular signaling. Our recent discovery that these reactions are differently balanced in animals and Arabidopsis prompted us to examine divergence throughout the lineage and evolution of the G proteins and 7TM-RGS proteins within the plant kingdom. To complement our phylogenetic analyses of plant signaling components, we purified an informative set of plant Gα proteins that spanned the plant kingdom (Figure 8), and also investigated an amino acid substitution that was deduced to have occurred in the grass ancestral Gα protein. All tested Gα subunits were able to release GDP quickly without any other regulatory protein such as a GPCR or other guanine nucleotide exchange factor (Table 2) (i.e. they were “self-activating”). This finding is consistent with the fact that no unequivocal homologous GPCR gene has been characterized in the plant kingdom [16], [17]. These results provide powerful evidence that plant G proteins use different regulatory mechanisms than vertebrates to activate and terminate G protein signaling.

We previously proposed that AtRGS1 functions as a sugar receptor GAP of AtGPA1, operating by a sugar-dependent GAP activity [6], [7]. Here we found that liverworts, representing non-vascular plants, lack RGS genes altogether. In compensation, the liverwort Gα hydrolyzes GTP to GDP quickly without the aid of an RGS protein (Figure 7*C* and Table 3). The rates of liverwort GDP-release and GTP-hydrolysis were each fast and similar in value (Table 2), suggesting that liverwort Gα activity is equally balanced between the two reactions. Thus, liverwort Gα protein is likely regulated by other proteins yet to be identified. In contrast to liverwort, OsRGA1 (representing monocots), shared similar intrinsic activation/inactivation properties with AtGPA1 (representing eudicots). This self-activating property of OsRGA1 was surprising given that all studied grass genomes lost the standard 7TM RGS gene. As with the nonvascular plants, this finding points toward alternative regulatory mechanisms in grasses that were not identified based on homology to known G protein regulators from animals. These results indicate that plant G proteins use at least three different regulatory mechanisms, not only to activate, but also to terminate G protein signaling.

In addition to GEFs, mammalian G proteins are also regulated by GDP dissociation inhibitor proteins (GDI), which inhibit GDP release from the G protein and stabilize the GDP-bound state [23]. Since all plant Gα subunits spontaneously release GDP (Table 2), and some lack RGS proteins, plant G proteins are likely regulated by molecules having GDI activity. While several proteins and chemicals have GDI activity [23], [24], to date no GDI has been found in the plant kingdom other than the Gβγ dimer, AGB1/AGG1, although this was shown to be insufficient to maintain Gα in the inactive state [25].

Our analyses also identified 7TM RGS gene loss in progress in the *S. italica* genome. It is not possible to determine whether the insertion of transposon-like elements found in the *S. italica* RGS gene actually caused loss of the 7TM domain function, or whether this functional loss predated the insertion events. Transposable elements are the most abundant DNA sequences coded in plant genomes, and confer rapid rearrangement of plant genome structure [26], [27]. It is interesting, however, that the *S. italica* RGS domain continues to be expressed and under purifying selection. The *S. italica* RGS protein could be specifically coupled with the *S. italica* Gα with the Asn substitution. However, whether it is still involved in G protein signaling, without the need for the 7TM domain, is not known.

The grass genomes that have been extensively sequenced are dominated by species that have been cultivated for centuries, including rice, sorghum, maize, barley, wheat and *S. italica* (aka, foxtail millet). However, the observed amino acid change in the Gα protein and the loss of a normal 7TM RGS is not an outcome of domestication per se, as we see the same Gα protein and 7TM genome structure in *S. viridis* (the wild ancestor of *S. italica*) (unpub. obs.). We have shown that deletion of AtRGS1 from Arabidopsis results in increased cell growth and proliferation [7], [8], [28], [29]. Also, Gα mutants in Arabidopsis and rice show defects in their development [28], [30]. Our analyses raise the intriguing possibility that G protein signaling regulates growth and development with different regulatory mechanisms in grasses and eudicots. Regulators other than 7TM-RGS await discovery, or the grass family could have divergent RH proteins not identified by BLAST search. In mammals, the Gs-class of α subunits lacks a known RGS protein. For the Gq-class of Gα subunits, phospholipase Cβ functions as a GAP of Gαq [31]. Therefore, it follows that G protein activity in grasses or the other plants may be regulated by divergent effecters or the other binding proteins yet to be identified in plants.

## Materials and Methods

### Collection of Gα, Gβ, Gγ, and 7TM-RGS sequences

The sequences of G protein signaling components were found using BLASTp (E value<0.1) against protein database and the translated BLAST (tBLASTn, E value<0.1) against genomic DNA sequences registered in Phytozome v7.0 (released on Apr/8/2011; www.phytozome.net) by using *A. thaliana* genes as queries. Full-length or partial DNA sequences of Gα, Gβ, Gγ, and 7TM-RGS for *Triticum aestivum*, *Hordeum vulgare*, *P. taeda*, *Picea glauca*, and *M.polymorpha* were identified with tBLASTn in the nucleotide collection (nr/nt) database or the expressed sequence tags (EST) database at National Center for Biotechnology Information (NCBI) or the species-specific EST database (E value<10). The partial DNA sequences were combined to determine the full cDNA sequences. G protein components of *P. dactylifera* were found using assembled-gene sequences downloaded from Weill Cornell Medical College in Qatar (http://qatar-weill.cornell.edu/research/datepalmGenome/download.html). 7TM-RGS gene of *P. dactylifera* was assembled manually. Gα genes of *P. taeda* and *P. glauca* were cloned from the cDNA libraries and their sequences were determined, because information from the databases was insufficient to define the full length sequence. To screen all the possible RGS-like genes, *P. dactylifera* RGS and *S. italica* RGS and RGS domain sequences from *H. sapiens* RGS4, G protein-coupled receptor kinase,, LARG and sorting nexin 13 were also used as query sequences (E value<10).

### Phylogenetic analysis

Phylogenetic trees were constructed with MEGA5.0 [32]. Full length Gα, Gβ and 7TM-RGS protein sequences were aligned with ClustalW using the following parameters; Gap opening penalty and gap extension penalty for initial pairwise alignment, 10 and 0.1; Gap opening penalty and gap extension penalty for multiple alignment, 10 and 0.2; Gonnet protein weight matrix; Residue-specific penalties, ON; Hydrophilic penalties, ON; Gap separation distance, 4; End gap separation, OFF; Use negative matrix, OFF. The maximum likelihood (ML) trees were made using the Complete-Deletion option of gaps and the JTT (Jones-Taylor-Thornton) substitution model [33] with gamma distributed rate variation, which was estimated as the best-fitting substitution model using MEGA5.0. The consensus phylogenetic trees were shown with the bootstrap values from 1000 repetitions. *Homo sapiens* Gα_i1_, Gα_q_, Gβ_1_ and Gβ_5_ were included as out groups.

### Plasmids and proteins

cDNAs of *P. taeda* RGS and Gα were amplified from the cDNA library and cloned into pENTR-D/TOPO vector. cDNAs corresponding to *O. sativa* or *M. polymorpha* Gα were synthesized with optimization of codon usage for *E.coli*. AtGPA1-T194N and OsRGA1-N191T mutants were created by site directed mutagenesis. The Gα cDNAs were subcloned into pDEST17 (N-terminal 6×His). Recombinant His-tagged Gα proteins were expressed in ArcticExpress RP (Agilent Technologies) or Rosetta(DE3) (Novagen, used only for PtGα1) with 0.5 mM IPTG at 12°C, solubilized in buffer A (50 mM Tris-HCl (pH 7.5), 100 mM NaCl, 5 mM MgCl_2_, 5 mM 2-Mercaptoethanol, 10 µM GDP, 1 mM PMSF and 1 µg/ml leupeptin) with 0.25 mg/ml lysozyme and 0.2% NP-40, captured from the soluble fraction with TALON Metal Affinity Resin (Clonetech), washed with buffer A containing 500 mM NaCl and 0.1% sodium cholate, and eluted with buffer A including 500 mM imidazole. 5 mM imidazole was added in crude extracts to reduce nonspecific binding. The purified proteins were dialyzed with buffer A and stored in 20% glycerol at −80°C. Recombinant His-AtRGS1 (284–459 aa) protein was prepared with the same method, except that MgCl_2_ and GDP were removed from buffer A.

### Gα activity

The rate of GTPγS binding was determined indirectly using intrinsic tryptophan fluorescence of Gα [20] and directly with [^35^S]GTPγS. The rate of GTP hydrolysis was determined with [γ^32^P]GTP.

For GTPγS binding, GDP-loaded Gα (1 µM) in TEDM buffer (50 mM Tris-HCl (pH 7.0), 1 mM EDTA, 1 mM DTT and 5 mM MgCl_2_) was mixed with an equal volume of TEDM buffer containing 5 µM [^35^S]GTPγS (about 5000 cpm/pmol) to start the binding reaction. At a given time points, 100 µl aliquots were quenched in 1 ml of ice-cold wash buffer (20 mM Tris-HCl (pH 7.5), 100 mM NaCl and 25 mM MgCl_2_) containing 50 µM GTP and immediately vacuum-filtered onto nitrocellulose. Filters were quickly washed three times with 3 ml of ice-cold wash buffer. The total amount of ^35^S bound to the filter was quantified by scintillation counting.

For single-turnover GTP hydrolysis reactions, Gα subunit (800 nM) was preloaded with radioactive [γ-^32^P]GTP in TEDL (50 mM Tris-HCl (pH 7.5), 10 mM EDTA, 1 mM DTT, and 0.05% lubrol-PX) for 30 min on ice. The hydrolysis reaction was then started by adding 450 µl of TMDL+GTPγS (50 mM Tris-HCl (pH 7.5), 40 mM MgCl_2_, 1 mM DTT, 0.05% lubrol-PX, and 400 µM GTPγS) into 1.2 ml of preloaded Gα. At a given time point, duplicate 100 µl aliquots were taken into 1 ml of charcoal (25% (w/v) in 50 mM phosphoric acid (pH 2.0)) to remove non-hydrolyzed [γ-^32^P]GTP and proteins. The charcoal tubes were centrifuged, and amount of ^32^PO_4_ hydrolyzed was measured by scintillation counting of the centrifuged supernatants.

GTP or GTPγS binding with Trp fluorescence and steady state GTP hydrolysis were performed as described previously [5], [25]. Briefly, 400 nM Gα protein was incubated in a cuvette with 1 ml of TEMNG buffer (25 mM Tris-HCl (pH 8.0), 1 mM EDTA, 5 mM MgCl_2_, 100 mM NaCl, and 5% glycerol). 800 nM GTP or 5 µM GTPγS was added to the cuvette and the change in the intrinsic fluorescence of Gα protein (excitation at 284 nm, emission at 340 nm) was monitored.

### *In vitro* binding

Affinity between 2 different proteins was measured by Surface Plasmon resonance technology using BIAcore 2000 (GE Healthcare). His-tagged AtRGS1 (284–459aa) was immobilized on sensor chip CM5 with ammine coupling. Temperature, flow rate or running buffer were 25°C, 10 µl/min, or 10 mM Hepes, 150 mM NaCl, 3 mM EDTA, 0.005% Tween-20, 100 µM GDP and 10 mM MgCl_2_, respectively. Seven different concentrations (6.25, 12.5, 25, 50, 100, 200 and 400 nM) of His-AtGPA1, AtGPA1-T194N, RGA1, RGA1-N195T or Gβγ (AGB1/AGG1) prepared in running buffer with 20 mM NaF and 100 µM AlCl_3_ were flowed onto the sensor chip for 3 min. Dissociation was monitored for 5 min, and the sensor chip was washed with the same running buffer for 10 min at a flow rate of 30 µl/ml. The association (k_a_) and dissociation (k_d_) rate constants were obtained by fitting the original sensorgrams with a 1∶1 Langmuir binding model.

## Supporting Information

Figure S1 Multiple alignments of plant Gα proteins. Full length amino acid sequences were aligned with ClustalW using following settings, gap opening penalty of 10 and gap extension penalty of 0.1 for initial pairwise alignment, gap opening penalty of 10 and gap extension penalty of 0.2 for multiple alignment, and Gonnet protein weight matrix. Three switch regions of Gα subunit are highlighted.
(PDF)

Figure S2 Multiple alignments of plant Gβ proteins. Full length amino acid sequences were aligned with ClustalW using following settings, gap opening penalty of 10 and gap extension penalty of 0.1 for initial pairwise alignment, gap opening penalty of 10 and gap extension penalty of 0.2 for multiple alignment, and Gonnet protein weight matrix.
(PDF)

Figure S3 Multiple alignments of plant RGS proteins. Full length amino acid sequences were aligned with ClustalW using following settings, gap opening penalty of 10 and gap extension penalty of 0.1 for initial pairwise alignment, gap opening penalty of 10 and gap extension penalty of 0.2 for multiple alignment, and Gonnet protein weight matrix. The transmembrane regions and RGS domains were highlighted with light green and orange. The transmembrane regions were predicted by SOSUI [Hirokawa et al, 1998] using *A. thaliana* RGS1. [Hirokawa T, Boon-Chieng S, Mitaku S (1998) SOSUI: classification and secondary structure prediction system for membrane proteins. Bioinformatics 14: 378–379.]
(PDF)

Figure S4 7TM-RGS gene in *P. dactylifera* (date palm). (A) Annotation details of *P. dactylifera* 7TM-RGS gene. The final gene model is within the green box. Dashed line green boxes show the positions of the 7TM and RGS domains. The published *P. dactylifera* annotation (light blue blocks) misses the 7TM domain. (B) The relationship of exons among 7TM-RGS genes in *Arabidopsis thaliana*, *P. dactylifera* and *V. vinifera* (grape). Exons are showed as blue blocks with introns as lines linking exons in the same gene. Lines linking exons in different genes indicate their homology. Block width reflects exons size, but intron sizes are only to scale in AtRGS1. This pattern indicates an intron loss between exon 6 and 7, leading to the fusion of two ancestral exons to form Arabidopsis exon 6. Checking orthologous gene structures in other dicots confirmed that the loss occurred after the divergence of the citrus and Arabidopsis lineages, and thus is shared by *A. thaliana* and *A. lyrata*.
(PDF)

Figure S5 Alignment of *S. italica* 7TM-RGS gene with AtRGS1. 7TM-RGS homologous sequences of *S. italica* were aligned with AtRGS1 protein. AtRGS1 is shown in gray. *S. italica* sequences found in plant GDB (http://www.plantgdb.org/SiGDB/, SiPROV019851m and SiPROV032159m) are highlighted with sky blue and green. Sequences found with BLAST search (Table S2) are shown in orange, yellow or purple.
(PDF)

Figure S6 RGS locus in *S. italic* (foxtail millet). (A) Annotation details of *S. italica* RGS gene. The final model is within the green box. This model is consistent with RGS gene annotation in Phytozome Setaria versions 6 and 7 (light blue boxes). The dashed line green box shows the position of the 7TM region, which is annotated as a gene in Phytozome v6, but not in Phytozome v7. This region does not have EST support. Two TEs, denoted as LTR-alubu and LINE, are detected between the *S. italica* RGS gene and the apparently pseudogenized 7TM domain. (B) Scope of the two TEs when using their closest known intact elements as references.
(PDF)

Table S1 G-protein components in the land plant kingdom. Homologous genes of *A. thaliana* AtGPA1, AGB1, and AtRGS1 were assembled from plant genome database through Phytozome v7.0 (released on Apr/8/2011; www.phytozome.net), nucleotide or EST database registered in NCBI (www.ncbi.nlm.nih.gov), and EST database of *M. polymorpha* (http://Marchantia.pmb.lif.kyoto-u.ac.jp). (1) All RGS genes were predicted to have 7- or 5-transmembrane domain, except non-transmembrane *S. italica* and *P. dactylifera* RGS. (2) Sequences of *G.max* G protein components were corrected according to previous research [13], because sequences registered in the soybean genome assembly (www.plantgdb.org/GmGDB/, Soybean Transcript (GenBank 170)) contain some deletions. (3) A Gγ homologous sequence is found highly in the *S. moellendorffii* genome (scaffold_123: 288795–289362 base) in JGI genome database, although the sequence has not assembled as Gγ gene. (4) A *P. patens* gene (XP_001772174.1) is incorrectly annotated as Gα in NCBI database. It is highly homologous to Arabidopsis extra-large GTP-binding protein (XP_002890957.1). (5) A *P. patens* Gγ gene is found in the EST and genome database, although it has not been assembled as a gene.
(PDF)

Table S2 BLAST search of *S.italica* 7TM-RGS gene. Genomic sequence of *S. italica* (Segment ID: 13, Bases: 1356001–1363646) was used as query for BLASTx against *A. thaliana* non-redundant protein sequences registered in NCBI. Results shown here are homologous sequences to AtRGS1 to fill a gap between SiPROV019851m and SiPROV032159m, which were similar to a part of 7TM-RGS gene.
(PDF)

Table S3 dn/ds values at 7TM and RGS domains. dN, the number of non-synonymous mutations per sites. dS, the number of synonymous mutations per sites.
(PDF)
