# Supplementary material for: G Protein Activation without a GEF in the Plant Kingdom
Source: PLoS Genet. 2012 Jun 28;8(6):e1002756. doi: 10.1371/journal.pgen.1002756 (PMC3386157; doi:10.1371/journal.pgen.1002756)
Supplement: Figure S1 — Multiple alignments of plant Gα proteins. Full length amino acid sequences were aligned with ClustalW using following settings, gap opening penalty of 10 and gap extension penalty of 0.1 for initial pairwise alignment, gap opening penalty of 10 and gap extension penalty of 0.2 for multiple alignment, and Gonnet protein weight matrix. Three switch regions of Gα subunit are highlighted. (PDF) [file pgen.1002756.s001.pdf]

Supplemental figure 1. Gα subunit

Arabidopsis thaliana GPA1	----------MGLLCSRSRHHT-EDTDENTQAAEIERRIEQEAKAEKHI-RKLLLLGAGESGKSTIFKQIKLLFQTGFDEGELKSYVPVIHANVYQTIK-
Arabidopsis lyrata	----------MGLLCSRSRHHT-EDTDENTQAAEIERRIEQEAKAEKHI-RKLLLLGAGESGKSTIFKQIKLLFQTGFDEGELKSYVSVIHANVYHTIK-
Ricinus comunis	--MLSSVVHNMGSLCSKQRRYNEADAEENAQAAEIERRIEQETKAEKHI-QKLLLLGAGDSGKSTIFKQIKLLFQSGFDESELKSYIPVIHANIYQTIK-
Manihot esculenta 1	--MLHIVIQNMGSLCSKQRRYSEADAEETAQAAEIERRIEQEAKAEKHI-QKLLLLGAGDSGKSTIFKQIKLLFQSGFDESELKSYISVIHANVYQTIK-
Manihot esculenta 2	--MLPIVIHNMGSLCSKQRRYKEADAEESAQAAEIERRIEQETKAEKHI-QKLLLLGAGDSGKSTIFKQIKLLFQSGFDESELKSYIPVIHANVYQAIK-
Populus trichocarpa 1	----------MGLLCSKRHRYNEADTEENAQAAEIERRIEQETKVEQHI-QKILLLGAGDSGKSTIFKQIKLLFQSGFDEAELKSYISVIHANVYQTIK-
Populus trichocarpa 2	----------MGSLCSKQRRCNEADAEENAQAAEIERRIEQETKAEKHI-QKLLLLGAGDSGKSTIFKQIKLLFQSGFDEAELKSYIPVIHANVYQTIK-
Mimlus guttatus	--MLCTLIDNMGLLCSR-HQHNQADSEENEQTAEIERRIEQETKSEKHI-QKLLLLGAGDSGKSTIFKQIKLLFQSGFDETELKGYIPVIHANVYQTIK-
Medicago truncatula	----------MGLLCSKSRRYNDANTEENAQTAEIERRIELETKAEKHI-QKLLLLGAGESGKSTIFKQIKLLFQTGFDEAELKSYLPVIHANVYQTIK-
Glycine max SGA2/GmGα2	--MLSFVTENMGLLCSRNRRYNDADAEENAQTAEIERRIELETKAEKHI-QKLLLLGAGESGKSTIFKQIKLLFQTGFDEAELKSYLPVIHANVYQTIK-
Glycine max GmGα3	--MLSFVTENMGLLCSRNRRYNDADAEESAQTAEIERRIELETKAEKHI-QKLLLLGAGESGKSTIFKQIKLLFQTGFDEAELKSYLPVIHANVYQTIK-
Glycine max SGA1/GmGα1	----------MGLVCSRSRRFREADAEENAQDAEIERRIELETKAEKHI-QKLLLLGAGESGKSTIFKQIKLLFQTGFDEAELKSYIPVVHANVYQTIK-
Glycine max GmGα4	----------MGLVCSRGRRFREADAEENAQDAEIERRIKLETKAEKHI-QKLLLLGAGESGKSTIFKQIKLLFQTGFDEAELKSYIPVIHANVYQAIK-
Vitis vinifera	----------MGSICSRHKHYHEADAEENAQAAEIERRIEQETKAEKHI-QKLLLLGAGESGKSTIFKQIKLLFQTGFDEAELKSYISVVHANVYQTIK-
Cucumis sativus	--MLSHLSRNMGLLCSRNRHYNEQDAEEKTQAAEIERRIEQETEAEKHI-QKLLLLGAGESGKSTIFKQIKLLFQTGFDEAELKSYIPVIHANVYQTIK-
Prunus persica	--MLSIVIENMGLLCSRNKHYNEADNEENAQTAEIERRIEQETKAEKHI-QKLLLLGAGESGKSTIFKQIKLLFQTGFDEAELKSYILVIHANVYQTIK-
Citrus sinensis	--MLSILIENMGLLCSKNRRYNAADSEENAQTAEIERRIEQETKAEKHI-QKLLLLGAGESGKSTIFKQIKLLFQTGFDEAELKSYISVIHANVYQTIK-
Citrus clementina	--MLSILIENMGLLCSKNRRYNAADSEENAQTAEIERRIEQETKAEKHI-QKLLLLGAGESGKSTIFKQIKLLFQTGFDEAELKSYISVIHANVYQTIK-
Eucalyptus grandis	--MLSSFIENMGLLCSRHRGYHDADAEENAQAVEIERRIEQETKAEKHI-QKLLLLGAGESGKSTIFKQIKLLFQTGFDEAELKSYVPVIHANVYQTIK-
Sorghum bicolor	MSVLTCVIESMGSSCSRSHSLDETEAAENAKSADIDRRILQETKAEQHI-HKLLLLGAGESGKSTIFKQIKLLFQTGFDEAELKSYTSVIHANVYQTIK-
Zea mays	MSVLTCVIESMGSSCSRSHSFDEAEAAENAKSADIDRRILQETKAEQHI-HKLLLLGAGESGKSTIFKQIKLLFQTGFDEAELRSYTSVIHANVYQTIK-
Setaria italica	MSVLTCVIESMGSSCSRHHSLNEAEAAENAKSADIDRRILQETKAEQHI-HKLLLLGAGESGKSTIFKQIKLLFQTGFDEAELRSYTSVIHANVYQTIK-
Brachypodium distachyon	MSVLTCVLESMGSSCSRPH-LNEAEAAENGKSAEIDRRILQETKAEQHI-HKLLLLGAGESGKSTIFKQIKLLFQTGFDEAELRSYISVIHANVYQTIK-
Triticum aestivum 1	----------MGSSCSRPHSVNEAEAADNTRSADIDRRILQETKADQHV-HKLLLLGAGESGKSTIFKQIKLLFRTGFDEAELKGYTPVIHANVFQTIK-
Triticum aestivum 2	----------MGSSCSRPHSVNEAEAADNTRSADIDRRILQETKADQHV-HKLLLLGAGESGKSTIFKQIKLLFRTGFDEAELKGYTPVIHANVFQTIKV
Triticum aestivum 3	----------MGSSCSRPHSVNEAEAADNTRSADIDRRILQETKADQHV-HKLLLLGAGESGKSTIFKQIKLLFRTGFDEAELKGYMPVIHANVFQTIK-
Hordeum vulgare	----------MGSSCSRPHSVNEAEAAGNTRSADIDRRILHETKADQHI-HKLLLLGAGESGKSTIFKQIKLLFRTGFDEAELKGYTPVIHANVYQTIK-
Oryza sativa RGA1	MSVLTCVLDNMGSSCSRSHSLSEAETTKNAKSADIDRRILQETKAEQHI-HKLLLLGAGESGKSTIFKQIKLLFQTGFDEAELRSYTSVIHANVYQTIK-
Phoenix dactylifera	----------MGSFCSRQKPYSEADAEENKQAAEIERRIAQETKAEQHI-HKLLLLGAGESGKSTIFKQIKLLFQTGFDEAELRSYTSVIHANVYQTIK-
Picea glauca	--MLSIFRQSMGSICSKQKPAAEEDDEE-CQAEEIDRRIAQEARAEKDV-QKLLLLGAGESGKSTIFKQIKLLFQTGFDEAERGNYISVIHANTYQSIK-
Pinus taeda PtGα1	--MLSIFRQSMGSLCSKQRPAAEEDSEE-RQAEEIDRRIAQEARAEKDV-QKLLLLGAGESGKSTIFKQIKLLFQTGFDEAERGNYISVIHANAYQSIK-
Marchantia polymorpha MpGα1	----------MGSVCGKDQQVSPRELEERVQAERIDQRIRRETKADKDVPKKLLLLGAGESGKSTIFKQIKVLFQEGFADGERINYKTVIYANVFQSMK-
Selaginella moellendorffii GPA-1	----------MGSLCSKGQPVAQEEDEE--RERELTRQLKQEDRIEKNV-YKLLLLGSGESGKSTIFKQIKLLYNTGFGVEELKNYTPVIHANVYQAIK-
Homo sapiens Gαq	----------MTLESIMACCLSEEAKEARRINDEIERQLRRDKRDARRE-LKLLLLGTGESGKSTFIKQMRIIHGSGYSDEDKRGFTKLVYQNIFTAMQ-
Homo sapiens Gαi1	----------------MGCTLSAEDKAAVERSKMIDRNLREDGEKAARE-VKLLLLGAGESGKSTIVKQMKIIHEAGYSEEECKQYKAVVYSNTIQSII-

Arabidopsis thaliana GPA1	------LLHDGTKEFAQNETDSAKYMLSSESIAIGEKLSEIGGRLDYPRLTKDIAEGIETLWKDPAIQETCARGNELQVPDCTKYLMENLKRLSDINYIP
Arabidopsis lyrata	------LLHDGTKEFAQNEADSAKYMLSSESIAIGEKLSEIGGRLDYPRLTKDLAEGIETLWKDPAIQETCARGNELQVPDCTKYLMENLKRLSDINYIP
Ricinus comunis	------ILHDGSKELAQNEADSSKYVISSENKDIGEKLSEIGGRLDYPCLTKELAKEIETLWKDDAIQETYGRGNELQVPDCAHYFMENLQRLSDANYVP
Manihot esculenta 1	------ILHDGSKELAQNETDSSKYVISSENKEIGEKLAEIGGRLDYPCLTKELAQETETLWKDAAIQETYIRGNELQVPDCAQYFMENLQRLSDPNYIP
Manihot esculenta 2	------ILHDGSKELAQNETDPSKYVISSENKDIGEKFSEIGGRLNYPCLTRDLAQEIETLWKDAAIQETYARGNELQVPDCAHYFMENLQRLSDANYIP
Populus trichocarpa 1	------VLHDGSKELAQNETDSLKYVISNENKDIGQKLSEIGGRLDHPSLTKELAQEIETLWRDAAIQETYARGNELQVPDCTPYFMENLQRLSDANYIP
Populus trichocarpa 2	------ILHDGSKELAQNETDSLKYVISNENKDIGKKLSEIGGRLDHPRLTKELAQEIETLWRDAAVQETYACGHKLQVPDCTPYFMDNLQRLSDSNYIP
Mimlus guttatus	------ILHDGSKELSLGSADSSDFIISDENKHLGEKFSEIGGRLDYPRLTKELAHEIETLWRDNAIQETYTRGNELQVPDCAHYFMENLQRLCDADYVP
Medicago truncatula	------LLHDGSKEFAQNDVDFSKYVISGENKDIGEKLSEIGGRLDYPRLTKELAQEIECLWKDPAIQETYSRGNELQVPDCTHYFMENLQRLSDANYVP
Glycine max SGA2/GmGα2	------LLHDGSKEFAQNDVDSSKYVISNENKEIGEKLLEIGGRLDYPYLSKELAQEIENLWKDPAIQETYARGSELQIPDCTDYFMENLQRLSDANYVP
Glycine max GmGα3	------LLHDGSKEFAQNDVDSSKYVISNENKEIGEKLSEIGGRLDYPYLTKELAQEIENLWKDPAIQETYARGSELQIPDCTDYFMENLQRLSDTNYVP
Glycine max SGA1/GmGα1	------VLHDGSKELAQNDFDSSKYVISNENQDIGEKLSEIGGRLDYPRLTKELAQEIETLWEDAAIQETYARGNELQVPDCAHYFMENLERLSDANYVP
Glycine max GmGα4	------VLHDGSKELAQNDVDSSKYVISHENQNIGEKLSEIGARLDYPCFTKELAQEIERLWEDAAIQETCARGNELQVPDCAHYFMENLERLSDANYVP
Vitis vinifera	------VLYDGSRELAQNTTDSSKYALSIENKDIGEKLSEIGGRLDYPRLTRELANEIETLWKDAAIQETYSRGNELQVPDCAHYFMDNLERLSDANYVP
Cucumis sativus	------VLHDGSKELAQNDKEFTKYVLSSENKDIGEKLSDIGGRLDYPRLTRERAQDIETLWKDAAIQETYSRGNELQVPDCTQYFMENLQRLSDANYIP
Prunus persica	------ILYDGSKELAQNNRE--TYEISSENKEIGEKLSKIGGRLDYPRLTKELAQDIETLWKDAAIQETYARGNELQVPDCANYFMENLQRLADANYIP
Citrus sinensis	------VLYDGSKELAQNETDSMKFVVSSENKEIGEKLSEIGGRLDYPRLTKELAEDIETLWADPAIQETYAHGNELQLPDCANYFMENLQRLSDANYVP
Citrus clementina	------VLYDGSKELAQNETDSMKFVVSSENKEIGEKLSEIGGRLDYPRLTKELAEDIETLWADPAIQETYAHGNELQLPDCANYFMENLQRLSDANYVP
Eucalyptus grandis	------ILYDGSKELAQNDGDSSKYIIADENKEIGEKLSEIGGRLDYPRLTKELAKEIESLWKDDAIQETFTCGYRFQVPDCAQYFMENLQRFSDVNYVP
Sorghum bicolor	------ILYEGAKELAQVEPDSSKYVLSPDSQEIGEKLSEIGVRLDYPSLNKECVQDVRKLWQDPAIQETYSRGSILQVPDCAQYFMENLDRLSEVDYVP
Zea mays	------ILYEGAKELAQVEPDSSKYVLSPDNQEIGEKLSEIGARLEYPSLNKERVQDVRKLWQDPAIQETYSRGSILQVPDCAQYFMENLDKLSEEDYVP
Setaria italica	------ILYDGAKELAQVEPDSSKYVLSPDNQEIGEKLSEIGAKLDYPLLNKELVQDVRKLWQDPAIQETYSRGSILQVPDCAQYFMSNLDRLAEVDYVP
Brachypodium distachyon	------ILYDGAKELAQVEPESSKYVISPDNQEIGEKISEIGGRLDYPLLCEELVHDIRKLWEDPAIQETYSRGSILQVPDCAQYFMENLDRLAEADYVP
Triticum aestivum 1	------ILYDGAKELAQLETESSKNVISPDNQEIGEKLSEIGGRLDYPLLNKELVQDVRKLWEDPAIQETYSCGSVLQVPDCAHYFMENLDRLAEPDYIP
Triticum aestivum 2	CQYWERILYDGAKELAQLETESLKHVISPDNQEIGEKLSEIGGRLDYPLLNKELVQDVRKLWEDPAIQETYSCGSVLQVPDCAHYFMENLDRLAEPDYIP
Triticum aestivum 3	------ILYDGAKELAQLETESSKHVISPDNQEIGEKLSEIGGRLDYPLLNKELVQDVRKLWEDSAIQETYSCGSVLQVPDCAHYFMENLDRLAEPDYIP
Hordeum vulgare	------ILYDGAKELAQVELESSKYVISSDNQEIGEKLSEIGGRLDYPLLNKELVQDVRKLWEDPAIQETYSCGSVLQVPDCAHYFMENLDRLAEADYVP
Oryza sativa RGA1	------ILYEGAKELSQVESDSSKYVISPDNQEIGEKLSDIDGRLDYPLLNKELVLDVKRLWQDPAIQETYLRGSILQLPDCAQYFMENLDRLAEAGYVP
Phoenix dactylifera	------ILYDGAKELGQNESDSSKYVVSIENKEIGEKLSAIGSRLDYPYLTKDIAHEIKTLWKDAAIQETYARGNILQVPDCAQYFMENLERLSEADYVP
Picea glauca	------ILYDGSKELAESEGEFSKYAISPEKKEIGEILSDIGGRPNYPPLTEKLAQDIEAVWKDNAIQETYLRANELQLPDCTHYFMENIHRLAQPNYMP
Pinus taeda PtGα1	------ILYDGSKELAESEGEFSKYAISPENKEIGEILSDIGGRPNYPPLTEKLAQDIATVWNDHAIQETYSRANELQLPDCTHYFMENIHRLAQPNYVP
Marchantia polymorpha MpGα1	------ILLDGLQEFAQ--SDQEKYTLKASNKAIGEELAEVGGRSELPLLTQDYAKKLELLWKDPAVQAAYARGSELQLPTCTEYFCNNLQRLSQIDYVP
Selaginella moellendorffii GPA-1	------ILYEGCLDLQKKDVSG-EYTMRRENMEHGKRIAEIGDGVDYHPIGLLESDLIAQIWSDPAIQATYRKANELQLPDCTEYFLSGVDRLAKPDYIP
Homo sapiens Gαq	-------------AMIRAMDTLKIPYKYEHNKAHA-QLVREVDVEKVSAFENPYVDAIKSLWNDPGIQECYDRRREYQLSDSTKYYLNDLDRVADPAYLP
Homo sapiens Gαi1	-------------AIIRAMGRLKIDFGDSARADDARQLFVLAGAAEEGFMTAELAGVIKRLWKDSGVQACFNRSREYQLNDSAAYYLNDLDRIAQPNYIP

Supplemental figure 1. Gα subunit

Arabidopsis thaliana GPA1	TKEDVLYARVRTTGVVEIQFSPVGENKKSGEVYRLFDVGGQRNERRKWIHLFEGVTAVIFCAAISEYDQTLFEDEQKNRMMETKELFDWVLKQPCFEKTS
Arabidopsis lyrata	TKEDVLYARVRTTGVVEIQFSPVGENKKSGEVYRLFDVGGQRNERRKWIHLFEGVTAVIFCAAISEYDQTLFEDEQKNRMMETKELFDWVLKQPCFEKTS
Ricinus comunis	TKEDVLYARVRTTGVVEIQFSPVGENKKSGEVYRLFDVGGQRNERRKWIHLFEGVTAVIFCAAISEYDQTLFEDENKNRMMETKELFEWVLKQPCFEKTS
Manihot esculenta 1	TKEDVLYARVRTTGVVEIQFSPVGENKKSGEVYRLFDVGGQRNERRKWIHLFEGVK---------LYDQTLFEDENKNRMMETKELFEWVLKQPCFEKTS
Manihot esculenta 2	TKEDILYARVRTTGVVEIQFSPVGENKKSGEVYRLFDVGGQRNERRKWIHLFEGVTAVIFCAAISEYDQTLFEDENKNRMVETKELFEWVLKQPCFEKTS
Populus trichocarpa 1	TKDDVLYARVRTTGVVEIQFSPVGENKKSGEVYRLFDVGGQRNERRKWIHLFEGVTAVIFCAAISEYDQTLFEDENKNRMIGTKELFEWVLKQPCFEKTS
Populus trichocarpa 2	TKEDVLYARVRTTGVVEIQFSPVGENKKSGEVYRLFDVGGQRNERRKWIHLFEGVTAVIFCAAISEYDQTLFEDENKNRMIETKELFEWVLKQPCFEKTS
Mimlus guttatus	TKEDVLYARVRTTGVVEIQFSPVGENKKSGEVYRLFDVGGQKNERRKWIHLFEGVSAVIFCAAISEYDQTLFEDDNKNRMMETKELLEWVLKQPCFEKTS
Medicago truncatula	TKEDVLLARVRTTGVVEIQFSPVGENKKSGEVYRLFDVGGQRNERRKWIHLFEGVSAVIFCVAISEYDQTLFEDENKNRMMETKELFEWVLKQQCFEKTS
Glycine max SGA2/GmGα2	TKEDVLYARVRTTGVVEIQFSPVGENKKSGEVYRLFDVGGQRNERRKWIHLFEGVSAVIFCAAISEYDQTLFEDENRNRMMETKELFEWILKQPCFEKTS
Glycine max GmGα3	TKEDVLYARVRTSGVVEIQFSPVGESKKSGEVYRLFDVGGQRNERRKWIHLFEGVSAVIFCAAISEYDQTLFEDENRNRMTETKELFEWILKQPCFEKTS
Glycine max SGA1/GmGα1	TKEDVLYARVRTTGVVEIQFSPVGENKRSGEVYRLFDVGGQRNERRKWIHLFEGVTAVIFCAAISEYDQTLYEDENKNRMMETKELFEWVLRQPCFEKTS
Glycine max GmGα4	TKEDVLYARVRTTGVVEIQFSPVGENKRSGEVYRLFDVGGQRNERRKWIHLFEGVTAVIFCAAISGYDQTLYEDENKNRMMETKELFEWVLKQPCFEKTS
Vitis vinifera	TKEDVLYARIRTTGVVEIQFSPVGENKKSGEVYRLFDVGGQRNERRKWIHLFEGVTAVIFCAAISEYDQTLFEDENKNRMMETKELFEWVLKQPCFQKTS
Cucumis sativus	TKEDVLYARVRTTGVVEIQFSPVGENKKSGEVYRLFDVGGQRNERRKWIHLFEGVTAVIFCAAISEYDQTLFEDEQKNRMMETKELFEWVLKQECFEKTS
Prunus persica	TKEDVLYARVRTTGVVEIQFSPVGENKKSGEVYRLFDVGGQRNERRKWIHLFEGVTAVIFCAAVSEYDQTLFEDESKNRMMETKELFDWVLKQPCFEKTS
Citrus sinensis	TKDDVLYARVRTTGVVEIQFSPVGEHKKSGEVYRLFDVGGQRNERRKWIHLFEGVSAVIFCAAISEYDQTLFEDEQKNRMMETKELFDWVLKQPCFEKTS
Citrus clementina	TKDDVLYARVRTTGVVEIQFSPVGEHKKSGEVYRLFDVGGQRNERRKWIHLFEGVSAVIFCAAISEYDQTLFEDEQKNRMMETKELFDWVLKQPCFEKTS
Eucalyptus grandis	TKDDVLYARVRTTGVVEIQFSPVGENKRSGEVYRLFDVGGQRNERRKWIHLFEGVSAVIFCAAISEYDQTLFEDENRNRMMETKELFDWVLKQPCFEKTS
Sorghum bicolor	TKEDVLHARVRTNGVVETQFSPLGESKRGGEVYRLYDVGGQRNERRKWIHLFEGVNAVIFCAAISEYDQMLCEDETKNRMMETKELFDWVLKQRCFEKTS
Zea mays	TKEDVLHARVRTNGVVETQFSPLGESKRGGEVYRLYDVGGQRNERRKWIHLFEGVNAVIFCAAISEYDQMLFEDETKNRMMETKELFDWVLKQRCFEKTS
Setaria italica	TKEDVLHARVRTNGVVETQFSPLGESKRGGEVYRLYDVGGQRNERRKWIHLFEGVNAVIFCAAVSEYDQMLFEDETKNRMMETKELFDWVLKQRCFEKTS
Brachypodium distachyon	TKEDVLHARVRTNGVVEIQFSPLGESKRGGEIYRLYDVGGQRNERRKWIHLFEGVDAVVFCAAISEYDQMLFEDEAQNRMMETKELFDWVLKQRCFEKTS
Triticum aestivum 1	TKEDVLHARVRTNGVVEIQFSPLGESKRGGEVYRLYDVGGQRNERRKWIHLFEGVDAAIFCAAISEYDQLLFEDGTQNRMMETKELFDWVLKQRCFEKTS
Triticum aestivum 2	TKEDVLHARVRTNGVVEIQFSPLGESKRGGEVYRLYDVGGQRNERRKWIHLFEGVDAVIFCAAISEYDQLLFEDETQNRMMETKELFDLVLKQRCFEKTS
Triticum aestivum 3	TKEDVLHARVRTNGVVEIQFSPLGESKRGGEVYRLYDVGGQRNERRKWIHLFEGVDAVIFCAAISEYDQLLFEDETQNRMMETKELFDWVLKQRCFEKTS
Hordeum vulgare	TKEDVLHARVRTNGVVEIQFSPLGESKRGGEVYRLYDVGGQRNERRKWIHLFEGVDAVIFCAAISEYDQLLFEDETQNRMMETKELFDWVLKQRCFEKTS
Oryza sativa RGA1	TKEDVLYARVRTNGVVQIQFSPVGENKRGGEVYRLYDVGGQRNERRKWIHLFEGVNAVIFCAAISEYDQMLFEDETKNRMMETKELFDWVLKQRCFEKTS
Phoenix dactylifera	TKEDVLYARVRTTGVVEIQFSPVGESRKSGEVYRLYDVGGQRNERRKWIHLFEGVTAVIFCAAISEYDQMLFEDETKNRMMETKELFDWVLKQACFEKTS
Picea glauca	TQDDVLYARVRTTGVAEIQFSPLGDNRRNGECYRLYDVGGQRNERRKWIHLFEGVTAVIFCAAISEYDQILFEDETKNRMMETKELFDWVLKQPWFEKTS
Pinus taeda PtGα1	TQEDVLYARVRTTGVAEIQFSPLGDNKRNGECYRLYDVGGQRNERRKWIHLFEGVTAVIFCAAISEYDQILFEDETKNRMMETKELFDWVLKQPWFEKTS
Marchantia polymorpha MpGα1	SQEDVLFARVRTSGIVETTFRPG-----RSDLYKLYDVGGQRNERKKWIHLFEGVTAVIFCAALSEYDQTLSEDENTNRMVEARDLFDWVLKQSCFKETS
Selaginella moellendorffii GPA-1	TEEDILHARVRTTGIADVVFKHDG------HTYRVFDVGGQRNERRKWLHLFDGVKAVIFCAALSEYDQNLFEDEGKNRMVETMELFESVLRHPSFEKTS
Homo sapiens Gαq	TQQDVLRVRVPTTGIIEYPFDLQS------VIFRMVDVGGQRSERRKWIHCFENVTSIMFLVALSEYDQVLVESDNENRMEESKALFRTIITYPWFQNSS
Homo sapiens Gαi1	TQQDVLRTRVKTTGIVETHFTFKD------LHFKMFDVGGQRSERKKWIHCFEGVTAIIFCVALSDYDLVLAEDEEMNRMHESMKLFDSICNNKWFTDTS

Arabidopsis thaliana GPA1	FMLFLNKFDIFEKKVLDVPLNVCEWFRDYQPVSSGK-QEIEHAYEFVKKKFEELYYQNTAPDRVDRVFKIYRTTALDQKLVKKTFKLVDETLRRRNLLEA
Arabidopsis lyrata	FMLFLNKFDIFEKKVLDVPLNVCEWFRDYQPVSSGK-QEIEHAYEFVKKKFEELYYQNTAPDRVDRVFKIYRTTALDQKLVKKTFKLVDETLRRRNLLEA
Ricinus comunis	FMLFLNKFDIFEKKILKVPLNVCEWFKDYQPVSTGK-QEIEHAYEFVKKKFEELYFQSTSPDRVDRVFKIYRTTALDQKLVKKTFKLVDETLRRRNLFEA
Manihot esculenta 1	FMLFLNKFDLFEKKVLKVPLNVCEWFKDYQPISTGK-QEIEHAYEFVKKKFEELYFQSTTPDRVDRVFKIYRTTALDQKLVKKTFKLVDETLRRRNLFEA
Manihot esculenta 2	FMLFLNKFDIFEKKVLKVPLSVCEWFKDYQPVSTGK-QEIEHAYEFVKKKFEELYYQSTTPDRVDRVFKTYRTTALDQKLVKKTFKLVDETLRRRNLLEA
Populus trichocarpa 1	FMLFLNKFDIFEKKVLKVPLNVCEWFKDYQPVLTGK-LEIEHAYEFVKKKFEELYFQSTTPDRVDRVFKIYRTTALDQKLVKKTFKLVDETLRRRNLFEA
Populus trichocarpa 2	FMLFLNKFDIFEKKVLKAPLNACEWFKDYQPISTGK-QEIEHAYEFVKKKFEELYFQSTTPDRVDRVFKIYRTTALDQKLVKKTFKLVDETLRRRNLFEA
Mimlus guttatus	FMLFLNKFDLFEKKVLQVPLNVCGWFKDYQPVSTGK-QEIEHAYEFVKKKFEELYFQSTAPDRVDRVFKIYRTTALDQKLVKKTFKLVDETLRRRNLFEA
Medicago truncatula	FMLFLNKFDIFEKKILDVPLNVCEWFKDYQPVSTGK-QEIEHAYEFVKKKFEESYFQNTAPDSVDRVFKIYRTTALDQKVVKKTFKLVDETLRRRNLFEA
Glycine max SGA2/GmGα2	FMLFLNKFDIFEKKILKVPLNVCEWFKDYQPVSTGK-QEIEHAYEFVKKKFEESYFQSTAPDRVDRVFKIYRTTALDQKVVKKTFKLVDETLRRRNLLEA
Glycine max GmGα3	FMLFLNKFDIFEKKILKVPLNVCEWFKDYQPVSTGK-QEIEHAYEFVKKKFEESYFQSTAPDRVDRVFKIYRTTALDQKVVKKTFKLVDETLRRRNLFEA
Glycine max SGA1/GmGα1	FMLFLNKFDIFEKKVLNVPLNVCEWFKDYQPVSTGK-QEIEHAYEFVKKKFEELYFQSTAPDCVDRVFKIYQATALDQKLVKKTFKLVDETLRRRNLFEA
Glycine max GmGα4	FMLFLNKFDIFEKKILNVPLNVCEWFKDYQPVSTGK-QEIEHAYEFVKKKFEELYFQSTAPDRVDRVFKIYRTTALDQKLVKKTFKLVDETLRRRNLFEA
Vitis vinifera	FMLFLNKFDIFEKKVIKVPLNVCEWFKDYQPVSTGK-QEIEHAYEFVKKKFEELYFQSTAPDCVDRVFKIYRTTALDQKLVKKTFKLVDETLRRRNLFEA
Cucumis sativus	FMLFLNKFDIFEKKVLKVPLSVCEWFNDYQPVSTGK-QEIEHAYEFVKKKFEELYFKSTAPDRVDRVFKVYRTTALDQKLVKKTFKLVDETLRRRNLFEA
Prunus persica	FMLFLNKFDIFEKKVLNVPLNVCEWFKDYQPVSTGK-QEIEHAYEFVKKKFEELYFQSTTPDRVDRVFKIYRTTALDQKLVKKTFKLVDETLRRRNLFEA
Citrus sinensis	FMLFLNKFDIFEKKVLKVPLNVCEWFKDYQPVSTGK-QEIENAYEFVKKKFEELYFQSTAPDRVDRVFKIYRTTALDPKLVKKTFKLVDETLRRRHLFEA
Citrus clementina	FMLFLNKFDIFEKKVLKVPLNVCEWFKDYQPASTGK-QEIEHAYEFVKKKFEELYFQSTAPDRVDRVFKIYRTTALDPKLVKKTFKLVDETLRRRHLFEA
Eucalyptus grandis	FMLFLNKFDIFEKKVLKVPLNVCEWFKDYESVSTGK-QEIEHAYEFVKKKFEELYYQCTAPERMDRVFKIYRTTALDQKLVKKTFKLVDETLRRRNLLEA
Sorghum bicolor	FMLFLNKFDIFERKIQKVPLSACEWFKDYQPIAPGK-QEVEHAYEFVKKKFEELYFQSSKPDRVDRVFKIYRTTALDQKLVKKTFKLIDESMRRSREGT-
Zea mays	FMLFLNKFDIFERKIQKVPLSVCEWFKDYQPTAPGK-QEVEHAYEFVKKKFEELYFQSSKPDRVDRVFKIYRTTALDQKLVKKTFKLIDESMRRSREGT-
Setaria italica	FMLFLNKFDIFERKIQKVPLSVCEWFKDYQPTAPGK-QEVEHAYEFVKKKFEELYFQSSKPDRVDRVFKIYRTTALDQKLVKKTFKLIDESMRRSREGT-
Brachypodium distachyon	FMLFLNKFDIFERKIQKVPLTVCDWFKDYQPIAPGK-QDVEHAYEFVKKKFEELYFQSSKPDRVDRVFKIYRTTALDQKLVKKTFKLIDESMRRSREET-
Triticum aestivum 1	FMLFLNKFDIFEREIQKVPLTVCEWFKDYEPIAPGKVQDVEHAYEFVKKKFEEVYFQSSKPERVDRVFKIYRTTALDQKLVKKTFKLIDESMRRSREGTG
Triticum aestivum 2	FMLFLNKFDIFERKIQKVPLTVCEWFKDYEPIAPGKVQDVEHAYEFVKKKFEEVYFQSSKPERVDRVFKIYRTTALDQKLVKKTFKLIDESMRRSREGTG
Triticum aestivum 3	FMLFLNKFDIFERKIQKVPLTVCEWFKDYEPIAPGK-QDVEHAYEFVKKKFEEVYFQSSKPDRVDRVFKIYRTTALDQKLVKKTFKLIDESMRRSREGTG
Hordeum vulgare	FMLFLNKFDIFERKIQKVPLTVCEWFKDYEPIAPGKVQDVEHAYEFVKKKFEEVYFQSSKPDRVDRVFKIYRTTALDQKLVKKTFKLIDESMRRSREGTG
Oryza sativa RGA1	FILFLNKFDIFEKKIQKVPLSVCEWFKDYQPIAPGK-QEVEHAYEFVKKKFEELYFQSSKPDRVDRVFKIYRTTALDQKLVKKTFKLIDESMRRSREGT-
Phoenix dactylifera	FMLFLNKFDIFEKKVQKVPLNVCEWFKDYQPKASGK-HEVEHAYEFVKKKFEELYFQSTKPDCVDRVFKIYRTTALDQKLVKKTFKLVDETLRRRNLIEA
Picea glauca	FLLFLNKFDIFETKVLKVPLNVCEWFRDYQPLTSGK-QEIEHAYEFVKKKFEELYFQNTPADRVDRVFKIYRTTALDQKLIKKTFKLVDETLTLRYLTDA
Pinus taeda PtGα1	FLLFLNKFDIFETKVLKVPLNVCEWFKDYKPLTSGK-QEIEHAYEFVKKKFEELYFQNTPADRVDRVFKIYRTTALDQKLIKKTFKLVDETLTLRYLTDA
Marchantia polymorpha MpGα1	FLLFLNKFDLFEQKIHKVPLSVCEWFSDYKPVSTGR-AEVSHAYQYVEKKFREVFHKNTNAGNVRRVFQVYRTTAVDKTLVEKTFNLVDEALTRELLSRG
Selaginella moellendorffii GPA-1	FLVFLNKYDIFRKKVLSVPLNVCEVFRDYNEVQGDQERKISHALQYIKNKFDEIYKRNTPGLGTQRLCWLFETTALDPRIMKYTFELVDKNLVVSSIS--
Homo sapiens Gαq	VILFLNKKDLLEEKIMYSHL------VDYFPEYDGPQRDAQAAREFILKMFVDLNPDSD------KIIYSHFTCATDTENIRFVFAAVKDTILQLNLKEY
Homo sapiens Gαi1	IILFLNKKDLFEEKIKKSPL------TICYPEYAGSNTYEEAA-AYIQCQFEDLNKRKDT-----KEIYTHFTCATDTKNVQFVFDAVTDVIIKNNLKDC

Supplemental figure 1. Gα subunit

Arabidopsis thaliana GPA1	G L L
Arabidopsis lyrata	G L L
Ricinus comunis	G L L
Manihot esculenta 1	G L L
Manihot esculenta 2	G L L
Populus trichocarpa 1	G L L
Populus trichocarpa 2	G L L
Mimlus guttatus	G L L
Medicago truncatula	G L L
Glycine max SGA2/GmGα2	G L L
Glycine max GmGα3	G L L
Glycine max SGA1/GmGα1	G L L
Glycine max GmGα4	G L L
Vitis vinifera	G L L
Cucumis sativus	G L L
Prunus persica	G L L
Citrus sinensis	G L L
Citrus clementina	G L L
Eucalyptus grandis	G L L
Sorghum bicolor	- - -
Zea mays	- - -
Setaria italica	- - -
Brachypodium distachyon	- - -
Triticum aestivum 1	T - -
Triticum aestivum 2	T - -
Triticum aestivum 3	T - -
Hordeum vulgare	T - -
Oryza sativa RGA1	- - -
Phoenix dactylifera	G L L
Picea glauca	G L L
Pinus taeda PtGα1	G L L
Marchantia polymorpha MpGα1	G F I
Selaginella moellendorffii GPA-1	- L L
Homo sapiens Gαq	N L V
Homo sapiens Gαi1	G L F
