# Supplementary material for: G Protein Activation without a GEF in the Plant Kingdom
Source: PLoS Genet. 2012 Jun 28;8(6):e1002756. doi: 10.1371/journal.pgen.1002756 (PMC3386157; doi:10.1371/journal.pgen.1002756)
Supplement: Figure S2 — Multiple alignments of plant Gβ proteins. Full length amino acid sequences were aligned with ClustalW using following settings, gap opening penalty of 10 and gap extension penalty of 0.1 for initial pairwise alignment, gap opening penalty of 10 and gap extension penalty of 0.2 for multiple alignment, and Gonnet protein weight matrix. (PDF) [file pgen.1002756.s002.pdf]

Supplemental figure 2. Gβ subunit

Arabidopsis thaliana AGB1	--MSVSE--LKERHAVATETVNNLRDQLRQRRLQLLDTDVARYSAAQGRTRVSFGATDLVCCRTLQGHTGKVYSLDWTPE-RNRIVSASQDGRLIVWNAL
Arabidopsis lyrata	--MSVSE--LKERHAVATETVNNLRDQLRQRRLQLLDTDVARYSAAQGRTRVSFGATDLVCCRTLQGHTGKVYSLDWTPE-RNRIVSASQDGRLIVWNAL
Citrus clementina	MSSSVSE--LKERHAAAIETVNSLRERLKQKRQQLLDVDVAGYSRSQGRAPVSFGPTDLVCCRTLQGHAGKVYSLDWTSE-RNRIVSASQDGRLIVWNAL
Citrus sinensis	MSSSVSE--LKERHAAAIETVNSLRERLKQKRQQLLDVDVAGYSRSQGRAPVSFGPTDLVCCRTLQGHAGKVYSLDWTSE-RNRIVSASQDGRLIVWNAL
Manihot esculenta 1	--MSVPE--LKARHVAATETVNSLRERLKQKRLLLLDTDVAGYARARGKSPISFGATDLVCCRTLQGHTGKVYSLDWTAE-KNRIVSASQDGRLIVWNAL
Ricinus comunis	--MSVAE--LKARHVAATETVNTLRERLKQKRLLLLDTDVAGYARAQGKTPVSFGPTDLVCCRTLQGHTGKVYSLDWTAE-KNRIVSASQDGRLIVWNAL
Manihot esculenta 2	--MSVAE--LKARHVAATETVNSLRERLKQKRLQLLDTDVAGYARGQGKSQVSFGATDLVCCRTLQGHTGKVYSLDWTPE-RNRIVSASQDGRLIVWNAL
Mimlus guttatus	--MSVAD--LKERHMAATETVNSLRDRLKQKRLLLLDTDVAGYARSQGKTPVTFGPTDLVCCRTLQGHTGKVYSLDWTPE-KNRIVSASQDGRLIVWNAL
Vitis vinifera	--MSVAE--LKERHMAATETVNSLRERLKQKRLLLLDTDVAGYARAQGKSPVTFGPTDLVCCRTLQGHTGKVYSLDWTTE-KNRIVSASQDGRLIVWNAL
Populus trichocarpa 2	--MSVSE--LKERHVAATETVNSLGERLKQKRLLLLDTDIAGHARAQGRNPVSFGPTDLVCCRILQGHTGKVYSLDWTPE-KNRIVSASQDGRLIVWNAL
Populus trichocarpa 1	--MSVSE--LKERHVAATETVNSLRERLKQKRLLLLDTDMARYARAQERSPVSFGPTDLVCCRTLQGHTGKVYSLDWTPE-RNRIVSASQDGRLIVWNAL
Eucalyptus grandis	--MSVAE--LKERHRAATETVNSLRERLKQKRVQLLDTDVAGYARTQGKTPVTFGATDLVCCRTLQGHTGKVYSLDWTPE-RNRIVSVSQDGRFIVWNAL
Cucumis sativus	--MSGSE--LKERHVAAIETVNSLKERLKQKRHSLLDTNVAGYARTLGRTPVSFGATDLVCCRTLQGHTGKVYSLDWTSE-RNRIVSASQDGRLIVWNAL
Prunus persica	--MSVAE--LKERHVAASETVNSLRERLKEKRASLLDTDVAGYAKSQGKTPLTFGPTDLVCCRTLQGHTGKVYSLDWTSE-KNRIVSASQDGRLIVWNAL
Glycine max GmGβ2	--MSVTE--LKERHSAATDTVNNLRHRLKQKRLSLLDTDVSGYARSQGRTPVTFGPTDLVCCRTLQGHTGKVYSLDWTSE-KSQIVSASQDGRLIVWNAL
Glycine max GmGβ1	--MSVTE--LKERHSAATDTVNNLRHRLKQKRLSLLDTDISGYARSQGRTPVTFGPTDLVCCRTLQGHTGKVYSLDWTSE-KSQIVSASQDGRLIVWNAL
Medicago truncatula	--MSVTE--LKERHIAATETVNNLRERLKQRRLSLLDTDIAGYAKSQGRTPVTFGPTDILCCRTLQGHTGKVYSLDWTSE-KNRIVSASQDGRLIVWNAL
Glycine max GmGβ3	--MSVTE--LKERHLAATETINSLRERLKERRLSLLDTDIAGYARSQGRAPVTFGPTDLVCCRALQGHTGKVYSLDWTSE-KNRIVSASQDGRLIVWNAL
Glycine max GmGβ4	--MSVTE--LKERHLAATETVNSLRERLKERRLSLLDTDIAGYARSQGRSPVTFGPTDLVCCRTLQGHAGKVYSLDWTSE-KNRIVSASQDGRLIVWNAL
Triticum aestivum	-MASVAE--LKEKHAAATASVNSLRERLRQRRQTLLDTDVEKYSKAQGRTAVSFNPTDLVCCRTLQGHSGKVYSLDWTPE-KNWIVSASQDGRLIVWNAL
Hordeum vulgare	-MASVAE--LKEKHAAATASVNSLRERLRQRRQTLLDTDVEKYSKAQGRTAVSFNPTDLVCCRTLQGHSGKVYSLDWTPE-KNWIVSASQDGRLIVWNAL
Brachypodium distachyon	-MASVAD--LKEKHAAATASVNSLRERLRQRRQLLLDTDVERYSKAQGRTAVSFNQTDLVCCRTLQGHSGKVYSLDWTPE-KNWIVSASQDGRLIVWNAL
Oryza sativa RGB1	-MASVAE--LKEKHAAATASVNSLRERLRQRRQMLLDTDVERYSRTQGRTPVSFNPTDLVCCRTLQGHSGKVYSLDWTPE-KNWIVSASQDGRLIVWNAL
Setaria italica	-MASVAE--LKEKHAAATASVNSLRERLRQRREMLLDTDVARYSKAQGRTPVSFNPTDLVCCRTLQGHSGKVYSLDWTPE-KNWIVSASQDGRLIVWNAL
Zea mays	-MASVAE--LKEKHAAATASVNSLRERLRQRRETLLDTDVARYSKSQGRVPVSFNPTDLVCCRTLQGHSGKVYSLDWTPE-KNWIVSASQDGRLIVWNAL
Sorghum bicolor	-MASVAE--LKEKHAAATASVNSLRERLRQRRETLLDTDVARYSKSQGRLPVSFNPTDLVCCRTLQGHSGKVYSLDWTPE-KNWIVSASQDGRLIVWNAL
Phoenix dacylifera	--MYVAE--LKERHMAATATVNGLRERLKQRRQALLDTDVAGYAKSHGKTAISFGSTDLVCCRTLQGHTGKVYSLDWTPE-RNRIVSASQDGRLIVWNAL
Picea glauca	--MSVAE--LRERHVAATAKVNALRERIKDKRRQLLDTDVATYASNNGRTPISFSFTDLVCCRTLQGHTGKVYSLDWTSE-KNRIVSASQDGRLIVWNAL
Pinus taeda	--MSVQE--LRERHAAATAKVNALRERIKAKRLQLLDTDVATYASSNGRTPISFSFTDLVCCRTLQGHTGKVYSLDWTSE-KNRIVSASQDGRLIVWNAL
Marchantia polymorpha	--MSNHD--VRERHAQAKAQVAGLRERLQESRSRKADTDVATYARGNGRSAVKFS-DDFRLCKTLQGHTGKIYSLDWAYQAEDRIVSASGDGRLIVWNAL
Selaginella moellendorffii	--MAGNGV-SQRDILGAQDEVDRLRSALQATRRRLQDTTLAEYARTHGRGDIGFAADRFKSRRMLSGHTGKVYALDWSER--DTIVSASQDGRLIVWNAM
Physcomitrella patens	--MSVADS-VRERYEVSKAEVQSLREQLHEKRASLLDTDVATTAQSQGCKPVDINNLYFKCVRTLQGHTGKIYSLDWAYRETSRIVSASQDGRLIVWNAI
Homo sapiens Gβ1	----------MSELDQLRQEAEQLKNQIRDARKACADATLSQITNN----IDPVGRIQMRTRRTLRGHLAKIYAMHWGTD-SRLLVSASQDGKLIIWDSY
Homo sapiens Gβ5	--MATEGLHENETLASLKSEAESLKGKLEEERAKLHDVELHQVAER----VEALGQFVMKTRRTLKGHGNKVLCMDWCKD-KRRIVSSSQDGKVIVWDSF

Arabidopsis thaliana AGB1	TSQKTHAIKLPCAWVMTCAFSPNGQSVACGGLDSVCSIFSLSSTADKDGTVPVSRMLTGHRGYVSCCQYVPNEDAHLITSSGDQTCILWDVTTGLKTSVF
Arabidopsis lyrata	TSQKTHAIKLPCAWVMTCAFSPNGQSVACGGLDSVCSIFSLSSTADKDGTVPVSRMLTGHRGYVSCCQYVPNEDAHLITSSGDQTCILWDVTTGLKTSVF
Citrus clementina	TSQKTHAIKLPCAWVMTCAFSPTGQSVACGGLDSVCSIFNLNSPTDKDGNLPVSRMLSGHKGYVSCCQYVPDEDTHLITSSGDQTCVLWDITTGLRTSVF
Citrus sinensis	TSQKTHAIKLPCAWVMTCAFSPTGQSVACGGLDSVCSIFNLNSPTDKDGNLPVSRMLSGHKGYVSCCQYVPDEDTHLITSSGDQTCVLWDITTGLRTSVF
Manihot esculenta 1	TSQKTHAIKLPCAWVMTCAFSPTGQSVACGGLDSVCSIFNLNSPIDKDGNLPISKMLSGHKGYISSCQYVPDEDTHLITSSGDQTCVLWDITTGLRTSVF
Ricinus comunis	TSQKTHAIKLPCAWVMTCAFSPTGQSVACGGLDSVCSIFNLNSPTEKDGNLPVSKMLSGHKGYVSACQYVPDEDTHLITSSGDQTCVLWDITTGLRTSVF
Manihot esculenta 2	TSQKTHAIKLPCAWVMTCAFSPTGQSVACGGLDSVCSIFNLNSPTDKDGNLPLSKMLSGHKGYVSSCQYVPDEDTHLITSSGDQTCILWDITTGLRTSVF
Mimlus guttatus	TSQKTHAIKLPCAWVMTCAFSPSGQSVACGGLDSMCSIFNLNSPTDKDGNLPVSRTLSGHKGYVSSCQYVPDEDTHLITASGDQTCVLWDITTGLRTSVF
Vitis vinifera	TSQKTHAIKLPCAWVMTCAFSPSGQSVACGGLDSVCSIFNLNSPTDKDGNLAMSKMLSGHKGYVSSCQYVPDEDTHLITSSGDQTCVLWDITTGLRTSVF
Populus trichocarpa 2	TSQKTHAIKLPCAWVMTCAFSPAGQSVACGGLDSVCSIFNLNSPTDKDGNLTVSRMLSGHKGYVSSCQYVPDEDTHLITSSGDQTCVLWDITTGLRTSVF
Populus trichocarpa 1	TSQKTHAIKLPCAWVMTCAFSPTGQSVACGGLDSVCSIFNLNSPTDKDGNLPVSRMLSGHKGYVSSCQYVPDEDTHLITSSGDQTCVLWDITTGIRTSVF
Eucalyptus grandis	TSQKTHAIRLPCAWVMTCAFAPNGQSVACGGLDSVCSIFNLNSPVDRDGNLPVSRMLSGHKGYVSSCQYVPDGDAHLITGSGDQTCVLWDITTGLRTSVF
Cucumis sativus	TSQKTHAIKLPCPWVMTCAFSPSGQSVAGGGLDSVCSIFNLNSPTDRDGNLPVTRTLCGHKGYISSCQYVPDEDTHLITGSGDQTCVLWDITTGLRTSVF
Prunus persica	TSQKTHAIKLPCAWVMTCAFSPTGQSVACGGLDSVCSIFNLNSLCDKDGNLPVSRTLSGHKGYVSSCQYVPDEDTHLITGSGDQTCVLWDITTGLRTSVF
Glycine max GmGβ2	TRQKIHAIKLPCAWVMTCAFSPTGQSVACGGLDSVCSIFNLNSPTDKDGNLPVSRMLSGHKGYVSSCQYVPDEDTHLITGSGDQTCVLWDITTGLKTSIF
Glycine max GmGβ1	TRQKIHAIKLPCAWVMTCAFSPTGQSVACGGLDSVCSIFNLNSPTDKDGNLPVSRMLSGHKGYVSSCQYVPDEDTHLITGSGDQTCVLWDITTGLKTSVF
Medicago truncatula	TSQKTHAIKLPCAWVMTCAFSPTGQSVACGGLDSVCSIFNLNSPTDRDGNLNVSRMLSGHKGYVSSCQYVPGEDTHLITGSGDQTCVLWDITTGLRTSVF
Glycine max GmGβ3	TSQKTHAIKLPCAWVMTCAFSPTGQSVACGGLDSVCSIFNLNSPADRDGNLAVSQMLSGHKGYVSSCQYVPDEDTHLVTGSGDQTCVLWDITTGFRTSVF
Glycine max GmGβ4	TSQKTHAIKLPCAWVMTCAFSPTGQSVACGGLDSVCSLFNLNSPTDRDGNLAVSRMLSGHKGYVSSCQYVPDEDTHLITGSGDQTCVLWDITTGLRTSVF
Triticum aestivum	TSQKTHAIKLHCPWVMECAFAPNGQSVACGGLDSACSIFNLSSQADRDGNMPVSRVLTGHKGYASSCQYVPDQETRLITGSGDQTCVLWDVTTGQRISIF
Hordeum vulgare	TSQKTHAIKLHCPWVMTCAFAPNGQSVACGGLDSACSIFNLSSQVDRDGNMPVSRVLTGHKGYVSSCQYVPDQETRLITGSGDQTCVLWDVTTGQRISIF
Brachypodium distachyon	TSQKTHAIKLHCPWVMTCAFAPNGQSVACGGLDSACSIFNLNSQVDRDGNMPVSRILTGHKGYVSSCQYVPDQETRLITGSGDQTCVLWDVTTGQRISIF
Oryza sativa RGB1	TSQKTHAIKLHCPWVMTCAFAPNGQSVACGGLDSACSIFNLNSQADRDGNIPVSRILTGHKGYVSSCQYVPDQETRLITSSGDQTCVLWDVTTGQRISIF
Setaria italica	TSQKTHAIKLHCPWVMTCAFAPNGQSVACGGLDSACSIFNLNSQADRDGNMPVSRILTGHKGYVSSCQYVPDQESRLITSSGDQTCVLWDVTTGQRISIF
Zea mays	TSQKTHAIKLHCPWVMACAFAPNGQSVACGGLDSACSIFNLNSQADRDGNMPVSRILTGHKGYVSSCQYVPDQETRLITSSGDQTCVLWDVTTGQRISIF
Sorghum bicolor	TSQKTHAIKLHCPWVMTCAFAPNGQSVACGGLDSACSIFNLNSQADRDGNMPVSRILTGHKGYVSSCQYVPDQETRLITSSGDQTCVLWDVTTGQRISIF
Phoenix dacylifera	TSQKTHAIKLYCAWVMTCAFAPNSQSVACGGLDSACSIFNLNSQVDRDGNIPVSRILTGHKGYVSSCQYVPDQETRLLTSSGDRTCVLWDVTTGQRISVF
Picea glauca	TSQKTHAIKLPCAWVMTCAFSPSGQAVACGGLDSVCSIFQLNSQIDRDGHLPASRILSGHRGYVSSCQYVPDGDTHVITGSGDRTCVQWDVTTGQRIAIF
Pinus taeda	TSQKTHAIKLPCAWVMTCAFSPSGQAVACGGLDSVCSIFQLNNQLDRDGHLPVSRILSGHRSYVSSCQYVPDGDTHVITGSGDRTCIQWDVTTGQRIAIF
Marchantia polymorpha	TSQKTHAIKLPCPWVMACAFSPRGRSVACGGLDNMCSIFNLSSPPDADGNLPVSQTLSGHKSYLSCCRYVPHSDNNIITSSGDETCILWDVDRSQKISTF
Selaginella moellendorffii	TSQKTHAIKLLCPWVMACAFSPDGETVACGGLDNLVSIFRLRS-FDSTGNHAPSAVLTGHKGYISCCRYVP-RKAQVLTSSGDHTCILWDVEAQRHIAQF
Physcomitrella patens	SSQKTYSLKLACAWVKAAAISPGGNTVACGGLDNVCSIFNLSSVPDKEGNLPVSGTLAGHTGYLSSCKYMPTQEKHIVTSSGDHTCRFWDVETQCCIAVF
Homo sapiens Gβ1	TTNKVHAIPLRSSWVMTCAYAPSGNYVACGGLDNICSIYNLKTR---EGNVRVSRELAGHTGYLSCCRFLD--DNQIVTSSGDTTCALWDIETGQQTTTF
Homo sapiens Gβ5	TTNKEHAVTMPCTWVMACAYAPSGCAIACGGLDNKCSVYPLTFDKN-ENMAAKKKSVAMHTNYLSACSFTNS-DMQILTASGDGTCALWDVESGQLLQSF

Supplemental figure 2. Gβ subunit

Arabidopsis thaliana AGB1	GGEFQSGHTADVLSVSIS-GSNPNWFISGSCDSTARLWDTRAASRAVR-TFHGHEGDVNTVKFFPDGYRFGTGSDDGTCRLYDIRTGHQLQVY-QPH--G
Arabidopsis lyrata	GGEFQSGHTADVLSVSIS-GSNPNWFISGSCDSTARLWDTRAASRAVR-TFHGHEGDVNTVKFFPDGYRFGTGSDDGTCRLYDIRTGHQLQVY-QPH--G
Citrus clementina	GGEFQSGHTADVLSVSIS-GSNSRMFVSGSCDSTARLWDTRVASRAVR-TFHGHEGDVNTVKFFPDGNRFGTGSDDGTCRLFDIRTGHQLQVYYQQH--G
Citrus sinensis	GGEFQSGHTADVLSVSIS-GSNSRMFVSGSCDSTARLWDTRVASRAVR-TFHGHEGDVNTVKFFPDGNRFGTGSDDGTCRLFDIRTGHQLQVYYQQH--G
Manihot esculenta 1	GGEFQSGHTADVLSVSIN-GSNLRMFVSGSCDSTARLWDTRVASRAVR-TFHGHEGDVNAVKFFPDGNRFGTGSDDGTCRLFDIRTGHQLQVYHQQH--R
Ricinus comunis	GGEFQSGHTADVLSVSIN-GSNSRMFVSGSCDSTARLWDTRVASRAVR-TFHGHEGDVSAVKFFPDGNRFGTGSDDGTCRLFDIRTGHQLQVYYQQH--N
Manihot esculenta 2	GGEFQSGHTADVLSVSIN-ASNSRMFVSGSCDSTARLWDTRVASRAVR-TFHGHEGDVNTVKFFPDGNRFGTGSDDGTCRLFDIRTGHQLQVYYQQH--S
Mimlus guttatus	GGEFQSGHTADVQSVSIN-GTNSRMFVSGSCDSTARLWDTRVASRAVR-TFHGHEGDVNTVKFFPDGNRFGTGSDDGTCRLFDIRTGHQLQEYSQPHS-G
Vitis vinifera	GGEFQSGHTADVLSVSIS-GSNSRMFVSGSCDATARLWDTRAASRAVR-TFHGHEGDVNTVRFFPDGNRFGTGSDDGTCRLFDIRTGHQLQVYYQEH--G
Populus trichocarpa 2	GGEFQSGHTADVLSVSIS-GSNSRMFVSGSCDSTARLWDTRVASRAVR-TFHGHEGDVNAVKFFPDGNRFGTGSDDGTCRLFDVRTGHQLQVYYQQH--G
Populus trichocarpa 1	GGEFQSGHTADVLSVSIN-GSNSRMFVSGSCDSTARLWDTRVASRAVR-TFHGHEGDVNAVKFFPDGNRFGTGSDDGTCRLFDIRTGHQLQVYYHQH--G
Eucalyptus grandis	GGEFQSGHTADVLSVSIN-GSSPRIFVSGSCDSTARMWDTRVASRAVH-TYHGHEGDVNAVKFFPDGNRFGTGSDDGTCRLFDIRTGHELQVYYQQR--G
Cucumis sativus	GGEFQSGHAADVSSISIS-GSNSRTFISGSCDATARLWDTRVASRAVQ-TFHGHEGDVSAVKFFPDGNRFGTGSDDGTCRLFDIRTGHELQVYHQLH--N
Prunus persica	GGEFQSGHTADVLSVSIN-QSNSRLFVSGSCDATARLWDTRVASRAVR-TFPGHEGDVNVVKFFPDGNRFGTGSDDGTCRLFDIRTGHQLQVYHQQH--G
Glycine max GmGβ2	GGEFQSGHTADVLSISIN-GSNSRMFVSGSCDATARLWDTRVASRAVR-TFHGHEGDVNAVKFFPDGNRFGTGSDDGTCRLFDIRTGHQLQVYYQQH--S
Glycine max GmGβ1	GGEFQSGHTADVLSISIN-GSNSRMFVSGSCDATARLWDTRVASRAVR-TFHGHEGDVNAVKFFPDGNRFGTGSDDGTCRLFDIRTGHQLQVYYQQH--S
Medicago truncatula	GGEFQSGHTADVLSISIN-GSNSKMFVSGSCDATARLWDTRVASRAVR-TFHGHEGDVNSVKFFPDGNRFGTGSEDGTCRLFDIRTGHQLQVYNQQHS-G
Glycine max GmGβ3	GGEFQSGHTADVLSISIN-GSNSRMFVSGSCDSTARLWDTRVASRAVR-TFHGHRGDVNTVKFFPDGNRFGTGSDDGTCRLFDIRTGHQLQVYHQQH--G
Glycine max GmGβ4	GGEFQSGHTADVLSISIN-GSNSRMFVSGSCDSTARLWDTRVASRAVQ-TFHGHQGDVNTVKFFPDGNRFGTGSDDGTCRLFDIRTGHQLQVYHRQH--G
Triticum aestivum	GSEFPSGHTADVLSLSIN-SLNANMFISGSCDTTVRLWDLRITSRAVR-TYHGHEGDINSVKFFPDGQRFGTGSDDGTCRLFDMRTGHQLQVYNREPDRN
Hordeum vulgare	GGEFPSGHTADVLSLSIN-SLNTNMFVSGSCDTTVRLWDLRIASRAVR-TYHGHEGDINSVKFFPDGQRFGTGSDDGTCRLFDMRTGHQLQVYNREPDRN
Brachypodium distachyon	GGEFPSGHTADVLSLSIN-PLNTNMFVSGSCDTTVRLWDLRIASRAVR-TYHGHEGDINSVKFFPDGQRFGTGSDDGTCRLFDMRTGHQLQVYNREPDRN
Oryza sativa RGB1	GGEFPSGHTADVLSLSIN-SSNSNMFVSGSCDATVRLWDIRIASRAVR-TYHGHEGDINSVKFFPDGQRFGTGSDDGTCRLFDVRTGHQLQVYSREPDRN
Setaria italica	GGEFPSGHTADVQSVSIN-SSNTNMFVSGSCDATVRLWDIRIASRAVR-TYHGHEADVNSVKFFPDGHRFGTGSDDGTCRLFDMRTGHQLQVYSREPNRD
Zea mays	GGEFPSGHTADVQSVSIN-SSNTNMFVSGSCDTTVRLWDIRIASRAVR-TYHGHEDDVNSVKFFPDGHRFGTGSDDGTCRLFDMRTGHQLQVYSREPDRN
Sorghum bicolor	GGEFPSGHTADVQSVSIN-SSNTNMFVSGSCDTTVRLWDIRIASRAVR-TYHGHEGDVNSVKFFPDGHRFGTGSDDGTCRLFDMRTGHQLQVYSRVPDRN
Phoenix dacylifera	GGEFPSGHTADVLSVSIN-SSNSNMFVSGSCDATARLWDTRIASRAVR-TYHGHVGDVNAVKFFPDGQRFGTGSDDGTCRLFDMRTGHQLQVYGQQHDNN
Picea glauca	GGEFPLGHTADVMSVSIS-AANPKEFVSGSCDTTTRLWDTRIASRAIR-TFHGHEADVNTVKFFPDGLRFGTGSDDGTCRLFDIRTGHQLQVYRQPP--R
Pinus taeda	GGEFPLGHTADVMSVSIS-AANPKEFVSGSCDTTTRLWDTRIASRAIR-TFHGHEADVNTVKFFPDGLRFGSGSDDGTCRLFDIRTGHQLQVYRQPP--R
Marchantia polymorpha	GGEATSGHTAAVMSVSVSTVSTPQVFVSGSCDKTARLWDTRMKS-APQ-TYQGHDGDVNAVCFLPDGKRFGTASDDGTCRLFDTRTGYQLQMYSHPR---
Selaginella moellendorffii	GSESSSGHTSDVMSLSIN-SQDPNQFVTGSCDTTAKLWDSRAPGRAAQCTFFSHTGDINAVQFLPDGCRFGTGSEDGICKLFDTRYGYQLQEYVTSG---
Physcomitrella patens	GGDILTGHTGDVMSVSVS-SSSPHVFISGSCDKSAKLWDVRTPARAQR-TFYGHEGDVNTVNFLSEGRHFGTGSDDGSCRLFDIGTGHELQQYWDKQALA
Homo sapiens Gβ1	TG-----HTGDVMSLSLAPDTR--LFVSGACDASAKLWDVREGMCRQT--FTGHESDINAICFFPNGNAFATGSDDATCRLFDLRADQELMTYSHDN---
Homo sapiens Gβ5	HG-----HGADVLCLDLAPSETGNTFVSGGCDKKAMVWDMRSGQCVQA--FETHESDINSVRYYPSGDAFASGSDDATCRLYDLRADREVAIYSKES---

Arabidopsis thaliana AGB1	DGENGPVTSIAFSVSGRLLFAGYAS--------NNTCYVWDTLLGEVVLDLG-LQQDSHRN--RISCLGLSADGSALCTGSWDSNLKIWA--FGGHRRVI
Arabidopsis lyrata	DGENVPVTSIAFSVSGRLLFAGYAS--------NNTCYVWDTLLGEVVLDLG-LQQDSHKN--RISCLGLSADGSALCTGSWDSNLKIWA--FGGHRRVI
Citrus clementina	ENEIPHVTSIAFSISGRLLFAGYS---------NGDCYVWDTLLAKVVLNLG-SLQNSHEG--RITCLGLSADGSALCTGSWDTNLKIWA--FGGHRRVL
Citrus sinensis	ENEIPHVTSIAFSISGRLLFAGYS---------NGDCYVWDTLLAKVVLNLG-SLQNSHEG--RITCLGLSADGSALCTGSWDTNLKVNLGFWRVQEGAL
Manihot esculenta 1	DNEVPQVTSIAFSVSGRLLFAGYS---------NGDCYVWDTLLAQVVLDLG-SLQNSHEG--RISCLGLSADGSALCTGSYDTNLKIWA--FGGHRKVI
Ricinus comunis	DNEVPHVTSIAFSISGRLLFAGYS---------NGDCYVWDTLLAQVVLDLG-SLQNSHEG--RISCLGLSADGSALCTGSYDTNLKIWA--FGGHRKVI
Manihot esculenta 2	DNEVPHVTSIAFSISGRLLFAGYT---------NGACYVWDTLLAQVVLDLG-SLQNSHQG--RISCLGLSADGSALCTGSYDTNLKIWA--FGGHRKVI
Mimlus guttatus	DSEPPQVTSIAFSISGRLLFAGYT---------NGACYVWDTLLAQVVLDLG-SLQNSHDGRSRISCLGLSADGSALCTGSYDTNLKIWA--FGGHRRVI
Vitis vinifera	DNEAPHVTSIAFSISGRLLFAGYS---------NGDCYVWDTLLAKVVLNLG-SLQNSHD--SRISCLGLSADGSALCTGSWDTNLKIWA--FGGHRRVI
Populus trichocarpa 2	DNEIAHVTSIAFSISGRLLFAGYS---------NGDCYVWDTLLAEVVLNLG-SLQNSHEG--RISCLGLSADGSALCTGSWDTNLKIWA--FGGHRKVI
Populus trichocarpa 1	DTEIPHVTSIAFSISGRLLFAGYS---------NGDCYVWDTLLAEAVLNLG-SLQNSHEG--RITCLGLSADGSALCTGSWDTNLKIWA--FGGHRQVI
Eucalyptus grandis	IDEIPHVTSIAFSISGRLLIAGYS---------NGDCFVWDTLLAQVVLNLG-SLQNSHEG--RISCLGVSADGSALCTGSWDTNLKIWA--FGGIRRVT
Cucumis sativus	DNTNSHVTSIAFSISGRLLFAGYK---------NGDCYVWDTLLAKMVLNLG-KLQNSHEG--RITCLGLSADGSALCTGSWDSNLKIWA--FGGHRKVT
Prunus persica	DNDFPPVKTIAFSISGRLLFAGYT---------NRDCYVWDTLLAKVVLNLG-SLQNSHEG--QISCLGLSADGSALCTGSWDTNLKIWA--FGGHRKII
Glycine max GmGβ2	DNDITPVTSIAFSASGRLLFAGYT---------NGDCYVWDTLLAKVVLNIG-SLQDSHED--RISCLGLSADGSALCTGSWDTNLKIWA--FGGHRRTT
Glycine max GmGβ1	DNEIPPVTSIAFSASGRLLFAGYT---------NGDCYVWDTLLAKVVLNIG-SLQDSHED--RISCLGLSADGSALCTGSWDTNLKIWA--FGGHRRTT
Medicago truncatula	DNEMAHVTSIAFSISGRLLIAGYT---------NGDCYVWDTLLAKVVLNLG-SLQNSHEG--RITCLGLSADGSALCTGSWDTNLKIWA--FGGHRKVI
Glycine max GmGβ3	DNEAAHVTSIAFSISGRLLFAGYT---------NGDCYVWDTLLAKVVLNLG-SLQNTHED--RISCLGLSADGSALCTGSWDTNIKIWA--FGGYRRVV
Glycine max GmGβ4	DNEAAHVTSIAFSMSGRLLFAGYT---------NGDCYVWDTLLAKVVLNLG-SLQNTHEG--RISCLGLSADGSALCTGSWDTNLKIWA--FGGYRRVN
Triticum aestivum	DNELPIVTSVAFSISGRLLFAGYS---------NGDCYVWDTLLAEMVLNLG-TLQNSHEG--RISCLGLSSDGSALCTGSWDKNLKIWA--FSGHRKIV
Hordeum vulgare	DNELPIVTSVAFSISGRLLFAGYS---------NGDCYVWDTLLAEVVLNLG-TLQNSHEG--RISCLGLSSDGSALCTGSWDKNLKIWA--FSGHRKIV
Brachypodium distachyon	DNELPIVTSIAFSISGRLLFAGYS---------NGDCYVWDTLLAEVVLNLG-TLQNSHDG--RISCLGLSSDGSALCTGSWDKNLKIWA--FSGHRKIV
Oryza sativa RGB1	DNELPTVTSIAFSISGRLLFAGYS---------NGDCYVWDTLLAEVVLNLG-NLQNSHEG--RISCLGLSSDGSALCTGSWDKNLKIWA--FSGHRKIV
Setaria italica	DNELPTVTSIAFSISGRLLFAGYS---------NGDCYVWDTLLAEVVLNLG-NLQNSHDG--RISCLGMSSDGSALCTGSWDKNLKIWA--FSGHRKIV
Zea mays	SNELPTVTSIAFSISGRLLFAGYS---------NGDCYVWDTLLAEVVLNLG-NLQNSHDG--RISCLGMSSDGSALCTGSWDKNLKIWA--FSGHRKIV
Sorghum bicolor	DDELPTVTSIAFSISGRLLFAGYS---------NGDCYVWDTLLAEVVLNLG-NLQNSHDG--RISCLGMSSDGSALCTGSWDKNLKIWA--FSGHRKIV
Phoenix dacylifera	DNDVPIVTSIAFSISGRLLFAGYS---------NGDCYVWDTLVAEVVLNLG-TLQNSHEG--RISCLGLSSDGSALCTGSWDKNLKIWG--FGGHRKVI
Picea glauca	ENQTPTVTSIAFSFSGRLLFAGYS---------NGDCFVWDTILEKVVLNLG-ELQNTHNN--RISCLGLSADGSALCTGSWDKNLKIWA--FGGHRRIA
Pinus taeda	ENQSPTVTAIAFSFSGRLLFAGYS---------NGDCFVWDTILEKVVLNLG-ELQNTHNG--RISCLGLSADGSALCTGSWDKNLKIWA--FGGHRKIV
Marchantia polymorpha	-GDSVKVLAVAFSSSGRLMFAAYD---------NEDCYVWDTIMSEVVTNLA-EHRHGHEK--RISCLGLSRDGSALCTGSWDNTLKVWA--FSGRRNRD
Selaginella moellendorffii	---NQLVTSIAFSHSGRILFAGYGNPTGVQNVVPTRCLLWDVLTATELHDIG----ESHSR--QVSCLGVAADGTALCTGSWDHTLKVWA--YSRTD---
Physcomitrella patens	RGPVN-VTSVAFSRSGRLLFAGYS---------NGNCYVWDTLLAKVVANLGGGRDDGHTN--RVSCLGLAADGDALCTGSYDATLKVWS--CAGKKS--
Homo sapiens Gβ1	--IICGITSVSFSKSGRLLLAGYD---------DFNCNVWDALKADRAGVLA-----GHDN--RVSCLGVTDDGMAVATGSWDSFLKIWN---------
Homo sapiens Gβ5	--IIFGASSVDFSLSGRLLFAGYN---------DYTINVWDVLKGSRVSILF-----GHEN--RVSTLRVSPDGTAFCSGSWDHTLRVWA---------

Supplemental figure 2. Gβ subunit

Arabidopsis thaliana AGB1	- - - - - - - -
Arabidopsis lyrata	- - - - - - - -
Citrus clementina	- - - - - - - -
Citrus sinensis	S P W G V K G L
Manihot esculenta 1	- - - - - - - -
Ricinus comunis	- - - - - - - -
Manihot esculenta 2	- - - - - - - -
Mimlus guttatus	- - - - - - - -
Vitis vinifera	- - - - - - - -
Populus trichocarpa 2	- - - - - - - -
Populus trichocarpa 1	- - - - - - - -
Eucalyptus grandis	- - - - - - - -
Cucumis sativus	- - - - - - - -
Prunus persica	- - - - - - - -
Glycine max GmGβ2	- - - - - - - -
Glycine max GmGβ1	- - - - - - - -
Medicago truncatula	- - - - - - - -
Glycine max GmGβ3	- - - - - - - -
Glycine max GmGβ4	- - - - - - - -
Triticum aestivum	- - - - - - - -
Hordeum vulgare	- - - - - - - -
Brachypodium distachyon	- - - - - - - -
Oryza sativa RGB1	- - - - - - - -
Setaria italica	- - - - - - - -
Zea mays	- - - - - - - -
Sorghum bicolor	- - - - - - - -
Phoenix dacylifera	H S L R - - - -
Picea glauca	- - - - - - - -
Pinus taeda	- - - - - - - -
Marchantia polymorpha	- - - - - - - -
Selaginella moellendorffii	- - - - - - - -
Physcomitrella patens	- - - - - - - -
Homo sapiens Gβ1	- - - - - - - -
Homo sapiens Gβ5	- - - - - - - -
