# Supplementary material for: G Protein Activation without a GEF in the Plant Kingdom
Source: PLoS Genet. 2012 Jun 28;8(6):e1002756. doi: 10.1371/journal.pgen.1002756 (PMC3386157; doi:10.1371/journal.pgen.1002756)
Supplement: Figure S3 — Multiple alignments of plant RGS proteins. Full length amino acid sequences were aligned with ClustalW using following settings, gap opening penalty of 10 and gap extension penalty of 0.1 for initial pairwise alignment, gap opening penalty of 10 and gap extension penalty of 0.2 for multiple alignment, and Gonnet protein weight matrix. The transmembrane regions and RGS domains were highlighted with light green and orange. The transmembrane regions were predicted by SOSUI [Hirokawa et al, 1998] using A. thaliana RGS1. [Hirokawa T, Boon-Chieng S, Mitaku S (1998) SOSUI: classification and secondary structure prediction system for membrane proteins. Bioinformatics 14: 378–379.] (PDF) [file pgen.1002756.s003.pdf]

Supplemental figure 3. RGS

Arabidopsis thaliana RGS1	--------MASG-CALHGGCPSDYVAVAISVICFFVLLSRS--VLPCLIHKAPRTNSSSFWIPVIQVISSFNLLFSIMMSVNLLRFRTKHWWRYCYLWAV
Arabidopsis lyrata	--------MASG-CAIHGGCPSDYVAVAISVICFFVLLSRS--VLPCLIHKAPRTNSSSFWIPVIQVIASFNLLFSIMMSVNLLRFRTKHWWRYCYLWAV
Citrus clementina	--------MAGSGCAVKGGCPTDYIALAIASLFFILLLARF--ILPFVVHKVPRTKSSGFWIPVIQVFASFNLLLSLVLSDNFLKFQRRHWWQSCYIWGV
Populus trichocarpa	MMKEEVNGSRS--CAVQGGCPSDYIAISIAILAFFLLLSRL--LFPFLIHKIPRTNGSGFWIPVIQVFGSFNLLLSIVMSINFLKFEKSHWWQSCYVWAV
Ricinus comunis	---MMSANVLKFRERHWWQSCYVWAV
Manihot esculenta	--------MAS--CAVEGGCATDYIAISISIISIILLLSRS--IFPFLVHKVPRINGSGFWIPLIQVFGSFNLLLSIVMSVNFLKFKKRHWWQSCYVWAV
Eucalyptus grandis	--------MAG--CAGEGGCPSDYVAVAISFLSLILLLYRS--ILPFVIHKFSRTKGSGFWIPVLQVFASFNLILSIVMSVDLLKFERRHWWQSCYVWAV
Cucumis sativus	--------MAR--CAVDGGCPSDYIAIVFAAVCLALLIFRW--ILPYVMYKIPLPKGSRFWIPVIQVFASLNLLLSIVISVSFFKFK-RQKWRSCYIWAV
Prunus persica	--------MAS--CAVYGGCASDYIAIAISAVFFILLLSRL--ILPFAIHKIPLPKRSSFWIPVIQIFASFNLLLSIVMSVNFLKFEKRHWWQSCYAWAV
Mimlus guttatus	--------MAT--CAVNGGCPSDYAAVSISLLSIFLLIGKA--VAPHLIHKIALPKGSSFWLVTIQIIASFNLIVSIVMSLGFLRFKRGHWWSSCYIWAV
Vitis vinifera	--------MGS--CGKNGGCPSDYIAVAISLLCFILLLIKA--TLPFLVHKVPRPKGSGFWIPVIQVFASLNLLLSIVMSINFLKFKKKHWWQSCYLWAV
Medicago truncatula	--------MANFKCAVKGGCPTDYVAVTVSILSFILLLIWS--IFPFIVHKVPRTKGSGFWIPVIQVVASFNLLLSIMMSHNFFKFEKSHWWQSCYLWAV
Glycine max RGS1	----------MVTCAVKGGCPSDYIAIALSILSITVLLLWS--IFPFLVHKVPRTKGSGFWLPVIQVVASFNLLLSIVMSNNFLKMGKRHWLRSCYLWGV
Glycine max RGS2	--MSNNFLKMGKRHWLRSCYLWGV
Phoenix dacylifera	--------------------MSFFLVNHLCHLPWFRLLARA--VFPFLVQKAPSTKASGLSLVIIQIIGSINLILSLVMSVNFLKWNQRQPWQSCYIWAV
Picea glauca	--------MMP--GYVPGGHPSDYVAVGFSILTLTLLIFLCVTAFAWNVHKVPKINTNHFQLLVIQTIGSISFLISLMMSINLLKFGHGHFWQSCYLWTV
Pinus taeda	--------MTP--KYVPGGHPSDYVAVGISILALTLIIFLCGTAFAWNVHKVPKIKTNHFQLLVIQTIGSITFLISLMMSVNLLKFGHGHFWQSCYLWTV
Selaginella moellendorffii	--------MTN--CRTVGGCVSDYVAAGITFFVIFLVCIGI--CFVSIKRRQLELNAKKVSVLLVQAFASLIYLVSSLMSLNYLRLSRRHLWQNCDLM-L
Setaria Italica	--

Arabidopsis thaliana RGS1	WIEGPLGFGLLMSCRITQAFQLYFIFVKKRLPPVKSYIFLPLVLLPWIFGAAIIHATKPLNDK---CHMGLQWTFPVAGLHALYVLALIAFTRAVRHVEF
Arabidopsis lyrata	WIEGPLGFGLLMGCRITQAFQLYFIFVKKRLPPIKSYIFLPLVLLPWIFGAAIIHATKPLNDK---CHMRLQWTFPVAGLHALYVLALIAFTRAVRHVEF
Citrus clementina	WIEGPLGFGLLMSCRIAQAFQLYYIFVRKHLPPIRSYVFLPLVLMPWLIAATFIQVMRPLNDR---CHMRVHWIIPFLFLHVVYVASLVGFMAAIRHIEF
Populus trichocarpa	WIEGPLGFGLLLSCRIAQAFQLHHIFVKRQLPPIRSYFFLPLILLPWVAGAAFVHVKRPLNDR---CHMGTHWIVPVVCLHTIYVAALVGFTWAIRHIEF
Ricinus comunis	WFEGPLGFGLLLSCRIAQAFQLYHIFVKRRLPPIRSYIFLPVILLPWIAGAAFIHVKKPLNAR---CHMETHWIIPVICLHASFIAALVGFTAAIRHIEF
Manihot esculenta	WFEGPLGFGLLLSCRITQAYQLYYIFVKRRLPLIRSYIFLPLILLPWIAGAAFIHVRKPLNTR---CHMGTHWIIPVVCLHIFYITSLVGFTGAIQHIEF
Eucalyptus grandis	WIEGPLGFGLLLSCRIVQAFQLYFIFAKRRLPPMRSHVCLPLFLLPWFAGAAFIHVKQPLNNH---CHMGAQWVVPVVSFHTLYVATLVAFTGAIRHIEF
Cucumis sativus	WVEGPLGFGLLLSSRITQTFQLYYIFVKRRLPPIKTHIFLPLILLPWISGAAFINVKKPLNYR---CHMGPRWIIPIMTLHILYVATLIVFTWAVRHIEF
Prunus persica	WIEAPLGFGLLLSCRISQAFQLYYIFVKRCLPPIRSYIFLPLILLPWIAGAALMHIKKPLNYR---CHMGTQWIIPVVSLHAIYVAILVGFTGAVRHIEF
Mimlus guttatus	WVEGPLGFGLLLSSRIVQAFQLYYIFVRRRLPPIRSFIFLPLVLLPWIIGAAFLHTKKPLNNR---CHMRTQWIIPVICLHALYVAAMVGFTAAIRHIEF
Vitis vinifera	WVEGPLGFGLLLSCRIVQAFQLYYIFVKRRLPPIRSYVFLPTIVLPWIAGAALLHKKKPLNER---CHLGTRWIIPVVLLHTTYVAALVGFTVAIRHIEF
Medicago truncatula	WGEGPLGFGLLLSSRITQAFQLYFIFVKRRLPLIRSFLLIPLILLPWIIGAAVIHIKKPLSNR---CHMSVQWTIPVVCLHALYVATLVGVTAAVHHIEF
Glycine max RGS1	WVEGPLGFGLLLSCRITQASQLYFIFVKRRLPLIRSYIFLPLILLPWIAAGGVIHSMKPLSSR---CHMKAQWTIPVVCLHSLYIAILVGVTAAVHHIEF
Glycine max RGS2	WVEGPLGFGLLLSCRITQASQLYFIFVKRRLPLIRSYIFLPLILLPWIAAGGVIHSMKPLSSR---CHMKAQWTIPVVSLHSLYIAILVGVTAAVHHIEF
Phoenix dacylifera	WAEGPLGFGLLMSCRIVQAFQLYHVFVKRRLPPVKPFVLLILILLPWIGGAAFIHINKPLNHQ---CHMRTQWVIPVVCIHAFYIAALIGVTRAVRHIEF
Picea glauca	WLEGPCGFGLLMSCRIAQAYRLYFLFVKKRLPVIKSYQLILLILLPWLGAAAFSQAKRPFNHF---CRVGNQWVIPIASLHVLYLSLLMGVTWRIRKIEF
Pinus taeda	WLEGPCGFGLLMSCRIAQAYRLYCLFVKRRLPVIKSYQLILLILLPWLGAAALSQAMRPFNHV---CHVGKQWVIPVASLHVLYLLVLMGVTWRIRKVEF
Selaginella moellendorffii	WLKGPFGFGILLSCHIVQAYRLHYVFIARRLPPLKYYQLLPLLVSPWFAVAAVIQAVSDHDKRSPTCQFNIRWELAVGSLYALYPAILLLATWKVRHIRF
Setaria Italica	--

Arabidopsis thaliana RGS1	RFDELRDLWKGILVSATSIVIWVTAFVLNEIH-EEISWLQVASRFVLLVTGGILVVVFFSISSNQPLLSQISLKKRQNFEF-QRMGQALGIPDSGLLFRK
Arabidopsis lyrata	RFDELRDLWKGILVSATSIIIWVTAFVLNEIH-EEISWLQVASRFVLLVTGGILVVVFFSISSNQPLLSQISLKKREHFEF-QRMGQALGIPDSGLLFRK
Citrus clementina	RFDELRDLWQGIIVSASSIGLWVFAYLLNEIH-DDISWLQVASRFLLLVMGGILVVVFFSISSSEPLLSQISLRKREPKEF-ETMGQALGIPDSGLLLRR
Populus trichocarpa	RFNELKDLWQGILVSSLSIGIWFAAYISNETR-DDIWWLQVASRILLLITASVLVLFFFSISSSQPLLSQISLRKREPLEF-QTMGQALGIPDSGLLLQI
Ricinus comunis	RFDELKDLWKGILVSASSIGLWVAAYILNEVH-DDVSWLQVLSRFLLLVTASILVLAFFSISSSEPLLSQISLRKREPLEF-ETMGQALGIPDSGVLLQR
Manihot esculenta	RFDELKDLWQGILVSSSSIGAWVVAYILNEIH-DDISWLQVISRFFLLTTASILVLVFFSISSSQPLLSQISLRKREPLQF-ETMGQALGIPDSGLLMQR
Eucalyptus grandis	RFNELRDLWRGIVLSASSIGVWIAAYVLNEIH-DEIAWLQVVSRFLLLVTSSILVLAFFSISSSQPLLSQISLRRREPLKY-ETMGQALGIPDSGLLMQR
Cucumis sativus	RFDELRDLWKGIIVSAFSIGVWVSAYITNEIH-EEILGLQVASRFLLLVTASILVLTFFSTSSSQPLLSQISLRKREALEY-DSMGHALGIPDSGLLLQR
Prunus persica	RFDELRDLWQGILVSASSIGVWVAAYILNEIH-DDISWLQVVSRFTLLVTASILVLSFFSISSSQPLLSQISLRKREPLAF-ETMGQALGIPNSGLLLPR
Mimlus guttatus	RFHELKDLWRGILVSTCSIGIWVIAYILNETH-EDIPLVQIVTRFLILVMTSVLVLVFFSISISQPLLSLMSLRKDEHQEYSMMMGRALGIPDSGLLVRT
Vitis vinifera	RFHELKDLWRGILVSTSSVGLWVTAYILNEIH-DDIEWLQVTSRFLLLIMASILVLAFFSMSSSQPLLSKMSLRKREATEF-ETMGRALGIPDSGLLLQR
Medicago truncatula	RFDELRDLWRGILVSSVSVAVWVTAYILNEIH-DNISWLQVVSRFLLLVLASILVLAFFSISSSQPLLSQISLRRRESREF-RTMGQALGIPDSGVLTQS
Glycine max RGS1	RFDELKDLWRGILVSVVSIAVWVTAYILNEIY-DNISWIEVASRFLLLVVASILVVAFFSISSSQPLLSQISLRRRESREF-RTMSQALGIPDSGVLAES
Glycine max RGS2	RFDELKDLWRGILVSAVSIAVWVTAYILNEIY-DNISWLEVASRFLLLVVASILVVAFFSISSSQPLLSQISLRRRESREF-RTMSQALGIPDSGVLAQS
Phoenix dacylifera	RFHEFKDLLQGIIVSSIAVGFWIGAYILNEVH-EDILWVQVVSRFLLLVTASVLVLVFFSMSVSQPLLSQISLRKRESTEF-VTMGQALGIPGCGLLVQT
Picea glauca	KFDEFKELSKGVAITLIFLGVWVSLYIANKVHGEKSTRLMVASRFTLMLAANILVLSFFFISMSRPLFTQLSMRKRESDQL-ETMGHVLGVPDSGLLFPT
Pinus taeda	KFDEFKELSKGVTITLIFIGVWVALYIANEVHGEKSTRLMVASRFMLMLAANILVLSFFFISMSRPLFTQLSVRKRESDKL-ETMGRILGVPESGLLFPT
Selaginella moellendorffii	EFDEFKELLKAASICALMLVVWAGMFVAAQLD------SKVVVTTRFIVAVSVGVLFLFWIPIAKPVHDLFHGRRSNHRYS--TMGQALGVPVSGAISQP
Setaria Italica	--MSISQPLHSQISLGKQESTAF-MTMGEALGITDRGS-VKK

Supplemental figure 3. RGS

Arabidopsis thaliana RGS1	EEFRPVDPNEPLDKLLLNKRFRHSFMEFADSCYAGETLHFFEEVYEHGKIPED-DSIRRIYMARHIMEKFIVAGAEMELNLSHKTRQEILTTQDLTHTDL
Arabidopsis lyrata	EEYRPVDPNEPLDKLLLNKRFRQSFMEFADSCYAGETLHFFEEVYEHGKIPVD-DSIRRIYMARHIMEKFIVSGAEMEVNLSHKTRQEILTTQDLTHSDL
Citrus clementina	EPTPVIDPNEPLDKLLLNKKFRQSFMAFADSCLAGESVHFFEEVHEHGKIPVD-DHVRRIYMARHIIEKYIVAGASMEVNISHRTRQEILTTSNLARPDL
Populus trichocarpa	DSVPVIDPNEPLDKLLLNKRFRQSFMAFADSCLAGESVHFYNEVHEHDKIPVD-DPVRRIYMARHIIEKYIVAGASMEVNISYRTRQEILTTTNLAHPDL
Ricinus comunis	DPAPVVDPNEPLDKLLQDKRFRQSFMAFADSCLAGENVHFYNEVHERGKIPLD-DTVRRIYMARHIIEKYIVAGAAMEVNISHRTREEILNTANLAHPDL
Manihot esculenta	DPAPVIDPNEPLDKLLLNKRFRQSFMDFADSCLAGESVHFYNEVHERGKIPID-DTIRRIYMTRHIIEKYIVAGAAMEVNISHRTRQEILTTVDLAHPDL
Eucalyptus grandis	EPSQAIDPDEPLDKLLLNKRFRQSFMAFADSCLAGESVHFHDDVHELGKIPVN-DSVRRIYMARDIIDKYITAGAPMEVNISYRCRQDILMTSNLAHPDL
Cucumis sativus	EPETVIDPNEPLEKLLLNKRFRRSFMAFADSCLAGENVHFYDEVHELGKLPLD-DPVRRIYMARHIIDNYITPGATMEVNISHRCRQEILTTSDLADPNL
Prunus persica	EPAPVIDPNEPLDKLLMNKRFRQSFMSFADSCLAGESVHFYEEVHELGKIPVD-DPVRRIYMARHIIDKYITAGATMEVNISHRSRQAILTTSNLAQPNL
Mimlus guttatus	ESLQSVDSNEPFDKLLSNRRFRQSFMEFADSCLAGESVHFYEEVQQLDRIPDS-DHVRRIYMARHIIDRYIAPGATMEVNISHRCRQEILSTLDLAHANL
Vitis vinifera	EPAPDIDPNEPLDKLLLNKGFRQSFMAFADSCLAGESVHFYDEVHELAKIPVD-DPVRRIYMARHIIEKYVIAGATMEVNISHRNRQEILTTPDLAHPDL
Medicago truncatula	EPISRVDPNEPLDKLLLNKKFRQSFMGFADSCLAGESVHFFDEVHELSKISEH-DCVRRIYMARHIIEKYMVAGAAMEINISHRSKQEILSTSDLARADL
Glycine max RGS1	EPISRIDPNEPLDKLLLNKRFRQSFMAFADSCLAGESVHFFDEVYELSKIPED-DCVKRIYMARHIIEKYIVAGAAMEVNISHRSRQEILSTSNLTRPDL
Glycine max RGS2	EPISRIDPNEPLDKLLLNKRFRLSFMAFADSCLAGESVHFFDEVYELSKIPED-DCVRRIYMARHIIEKYIIAGVAMEVNISHRSRQEILSTSSLTRPDL
Phoenix dacylifera	ASVAGVDLHQPLDKLLQDKRFRQSFMDFADSCLAGESVHFFEEVHELGKIPLD-DPVRRIYMARHIIENYIVTGAEMEINISHRTRQEILGTLDLAHPDL
Picea glauca	G--MPLNLDEPLEKLLRQKIFRQSFMAFADSRMAGESVHFYDEVHELNKIPIG-DTARRIYMAGHIIEQYIAVDSPMEINISHQMRQEILNTTDLAHPDL
Pinus taeda	G--MPLNLDEPLEKLLRQKKFRESFMAFADSRMAGESVHFYDEVHELNKIPTG-DTARRIYMAGHIIEQYIAVDSPMEINISHQMRQEILDTTDLAHPDL
Selaginella moellendorffii	LIAFNFN--RPLELLLEHRRFRQSYLAFADSRMAGEVVHFYSEVRELNKLQQQHQVVQRIYMAEHIIDKYIKSGAPMEINISHQMRLDIINTTDLADPDL
Setaria Italica	INATHFDSNQPLDKLLEDKRFRMSFMSFADSCLAGESVHFYEEVYDLKKIHLD-DSIRRIYMARHIIEKYIDAGAEMEINISHRTRQEILGTPDLTHPNL

Arabidopsis thaliana RGS1	FKNALNEVMQLIKMNLVRDYWSSIYFIKFKEEE----SCHEAMHKEGYSF------------SSPRLSSVQGSDDPFYQEHMSKSSRCSSPG--------
Arabidopsis lyrata	FKNALNEVMQLIKMNLVRDYWTSIYFIKFKEEE----SCDEAMQKEGWSF------------SSPRLSSVQGSDDPFYQEHMSKSSRCSSPG--------
Citrus clementina	FNNAINELMQLVKMNLAKDYWSSMFFIKLKEEASMRATGHELEQM-GWN-------------FSPRWSSVHCADDPFHDEQFSKGSEHSSHDLDP-----
Populus trichocarpa	FNNALNELMQLMKTNLVKDYWSSMFFIKFK-EANMRFNGHEQESMAGWN-------------FSPRLSSVRGADDPFHQEHVLKGFGCDNRDLDTQ----
Ricinus comunis	FNKAINELLQLMKTNLAKDYWSSMFFIKFKEEASLRSHSHDPEQMAVWN-------------FSPRLSSVHGLDDPFHQEPHVKGSGCTGHDLDARCQEL
Manihot esculenta	FNKAINELLQLMKMNLGKDYWTSMFFIKFKEEANMRSSDHDLEQMAGWN-------------FSPRLSSVHGADDPFHQEHPVKDTGCNNHGSDTRSQEL
Eucalyptus grandis	FNNALNELLQLMKMNLAKDYWSSTFFLKFKEEASMRLDAHEMEPMTGWN-------------FSPRLSGVRGADDPFHQEYLTKDSDHSSGNTDAT----
Cucumis sativus	FNNALNELIQLIKMNLAKDFWSSMFFLKLKEETSMRSNGRDLEQMASWN-------------LSPRLSSVQGTDDPFNQEQFSKGSGHDSTHDSDH----
Prunus persica	FNDALNELIQLMKMNLANDYWSSTYFMKFKEEASLRS--HELQQMTSWNS------------PTPRLSSVRGVDDPFHQEQEP-----------------
Mimlus guttatus	FKNALIELIHLMKMNLANDYWSSTFYMKLKDEAAMKTVDHELETG-GWN-------------FSQRLSSVHCTDDPFHQEHSPRKLGSSTNRDTELQLRE
Vitis vinifera	FNNALNELLQLMKMNLAKDYWSSMFFKKLR-EDGTGSNGHELEVVTGWN-------------YSPRLSCVHGEDDPFHEEHPSKSSAHDTNTQDVEQQ--
Medicago truncatula	FHNALNEIVHLMKTNLAKDYWSSMFFLKFQEECDMRCNGYELEQMTGWN-------------YSPRLSSVHGTDDPFHHDHLLKNSECGNDTDS------
Glycine max RGS1	FHNALNEIIQLMRTNLARDYWSSMFFLKFQEDSNVRSNEYELEQITGWN-------------FSPRLSSVHGTDDPFHQDHLLKSSGCSNDTTNL-----
Glycine max RGS2	FHNALNEIIQLMKTNLARDYWSSMFFLKFQEDTNVRSNEYELEQMTGWN-------------FSPRLSSVHGIDDPFHQDHLLKSSGCSNDTTDL-----
Phoenix dacylifera	FNHAVNEMMQLMKMNLLKDYWSSMNFVKFKEENPRQPDSSEP---AGWD-------------LSPRLSCVRCTDNPFNHEQLHKCPSGRKCDV-------
Picea glauca	FTNAVNEMVRMMQMNLEKDYWNSTFYSRFKEEIKDVAEASDVWDNTTIWD------------YSPKVSFVHGTDDPFDQDSLCRNSDGSWQSSMKNNNAY
Pinus taeda	FTNVVNEMVRMMQMNLEKDYQNSTFYSRFKEEIRDAAEACELWDNTTIWD------------YSPKVSFVHGTDDPFDHDSLCRNSEG------------
Selaginella moellendorffii	FKRAVGEVLKMMQMNLSNDYWQSEYFQELKDELDADVESQQTGDDLEQQLDVTATKRALRRLYDPTPDNPFDQGCPFSGELRKENQPEQHHTGESQSQRS
Setaria Italica	FDSAVSEILQLIKMNLAKDYWSSLHFAKLKEDIERGPNGPEL---MPLD-------------YSPRVTFVRCTDDPFYEEHAATCN--------------

Arabidopsis thaliana RGS1	--------------
Arabidopsis lyrata	--------------
Citrus clementina	--------------
Populus trichocarpa	--------------
Ricinus comunis	--------------
Manihot esculenta	--------------
Eucalyptus grandis	--------------
Cucumis sativus	--------------
Prunus persica	--------------
Mimlus guttatus	KFP-----------
Vitis vinifera	--------------
Medicago truncatula	--------------
Glycine max RGS1	--------------
Glycine max RGS2	--------------
Phoenix dacylifera	--------------
Picea glauca	P-------------
Pinus taeda	--------------
Selaginella moellendorffii	EILLTSQDTTLPRS
Setaria Italica	--------------
