# Supplementary figures and images for: G Protein Activation without a GEF in the Plant Kingdom
Source: PLoS Genet. 2012 Jun 28;8(6):e1002756. doi: 10.1371/journal.pgen.1002756 (PMC3386157; doi:10.1371/journal.pgen.1002756)

Supplemental figure 4

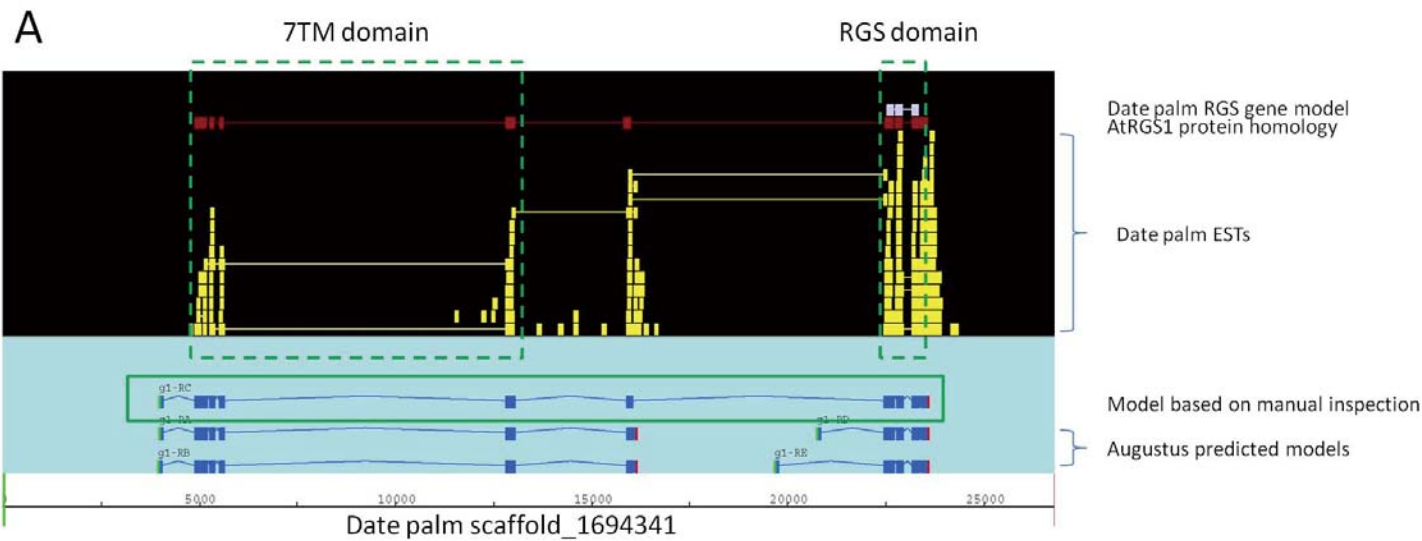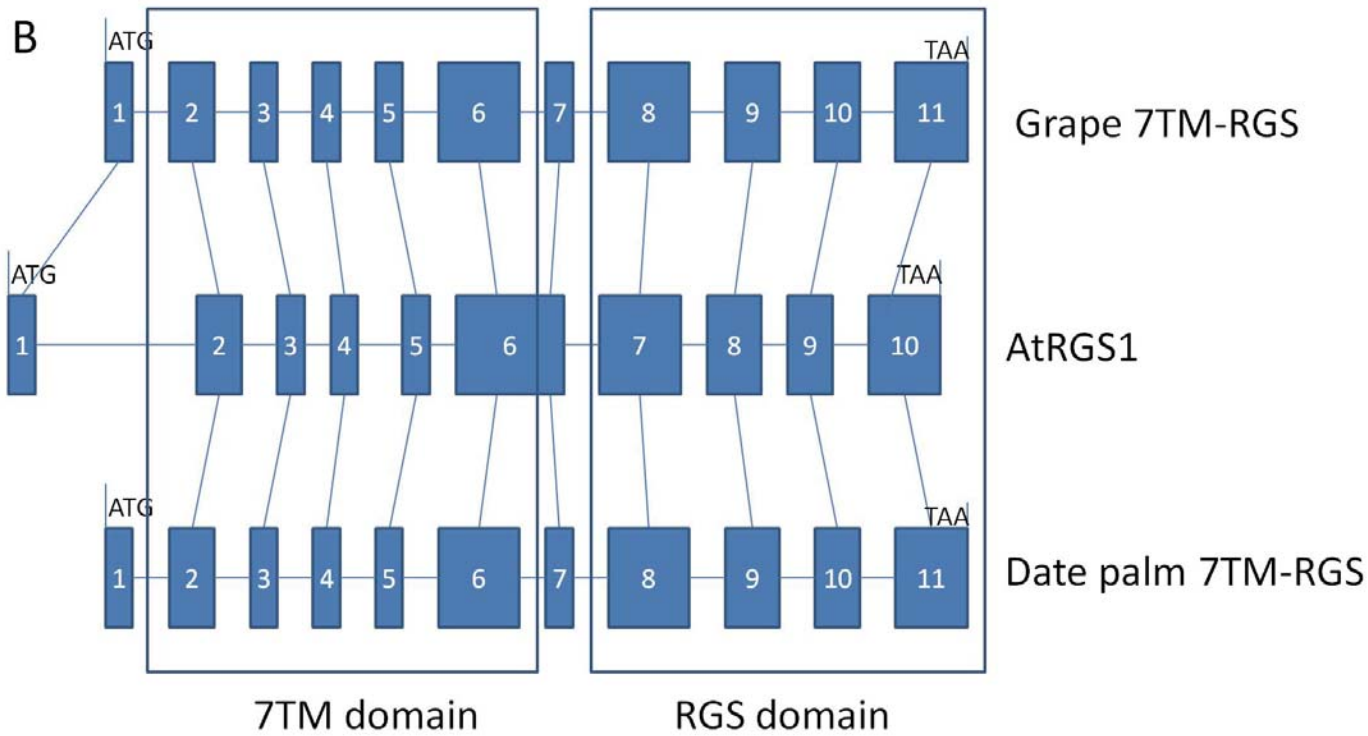

Supplement: Figure S4 — 7TM-RGS gene in P. dactylifera (date palm). (A) Annotation details of P. dactylifera 7TM-RGS gene. The final gene model is within the green box. Dashed line green boxes show the positions of the 7TM and RGS domains. The published P. dactylifera annotation (light blue blocks) misses the 7TM domain. (B) The relationship of exons among 7TM-RGS genes in Arabidopsis thaliana, P. dactylifera and V. vinifera (grape). Exons are showed as blue blocks with introns as lines linking exons in the same gene. Lines linking exons in different genes indicate their homology. Block width reflects exons size, but intron sizes are only to scale in AtRGS1. This pattern indicates an intron loss between exon 6 and 7, leading to the fusion of two ancestral exons to form Arabidopsis exon 6. Checking orthologous gene structures in other dicots confirmed that the loss occurred after the divergence of the citrus and Arabidopsis lineages, and thus is shared by A. thaliana and A. lyrata. (PDF) [file pgen.1002756.s004.pdf]

## Supplemental figure 5

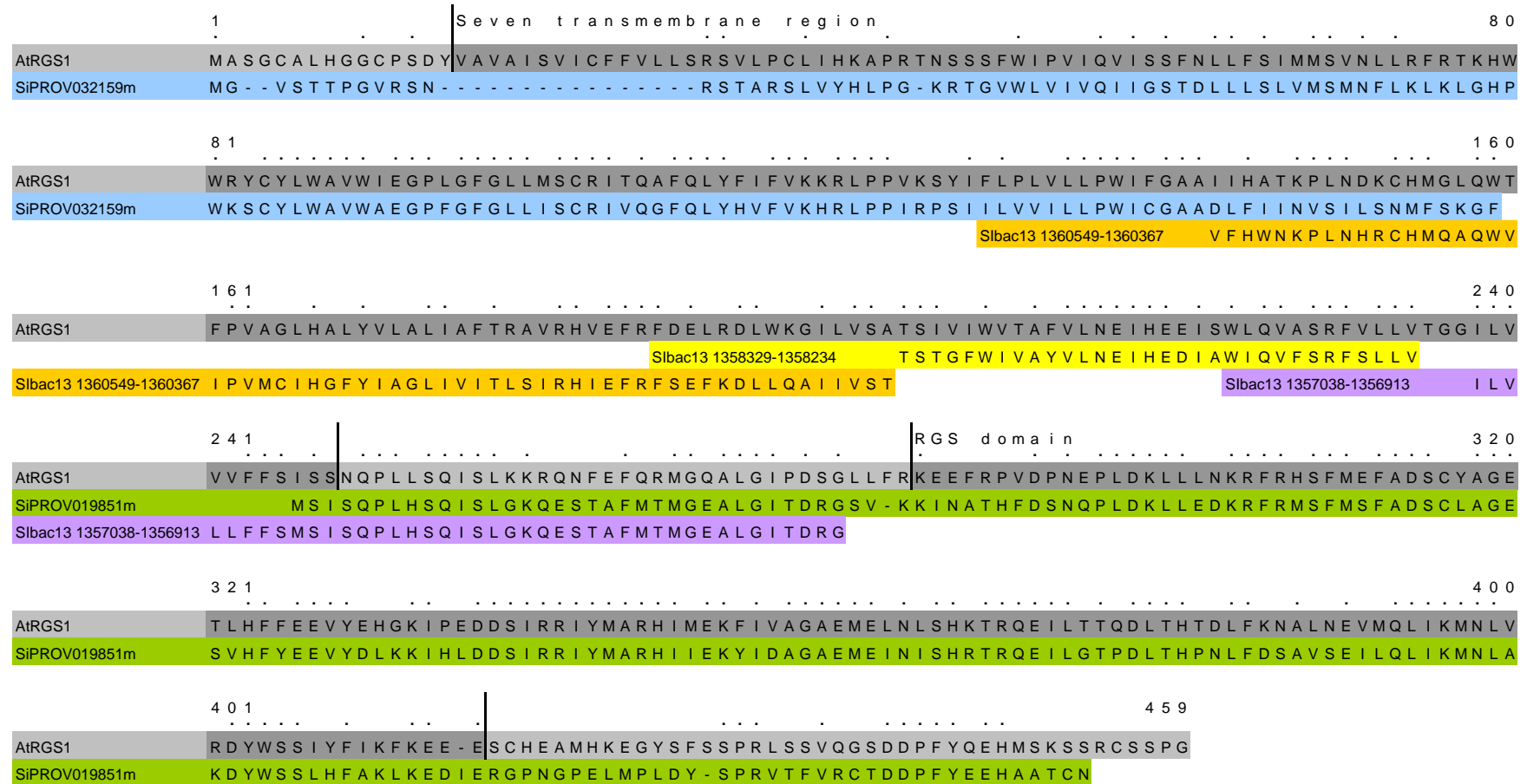

Supplement: Figure S5 — Alignment of S. italica 7TM-RGS gene with AtRGS1. 7TM-RGS homologous sequences of S. italica were aligned with AtRGS1 protein. AtRGS1 is shown in gray. S. italica sequences found in plant GDB (http://www.plantgdb.org/SiGDB/, SiPROV019851m and SiPROV032159m) are highlighted with sky blue and green. Sequences found with BLAST search (Table S2) are shown in orange, yellow or purple. (PDF) [file pgen.1002756.s005.pdf]

Supplemental figure 6

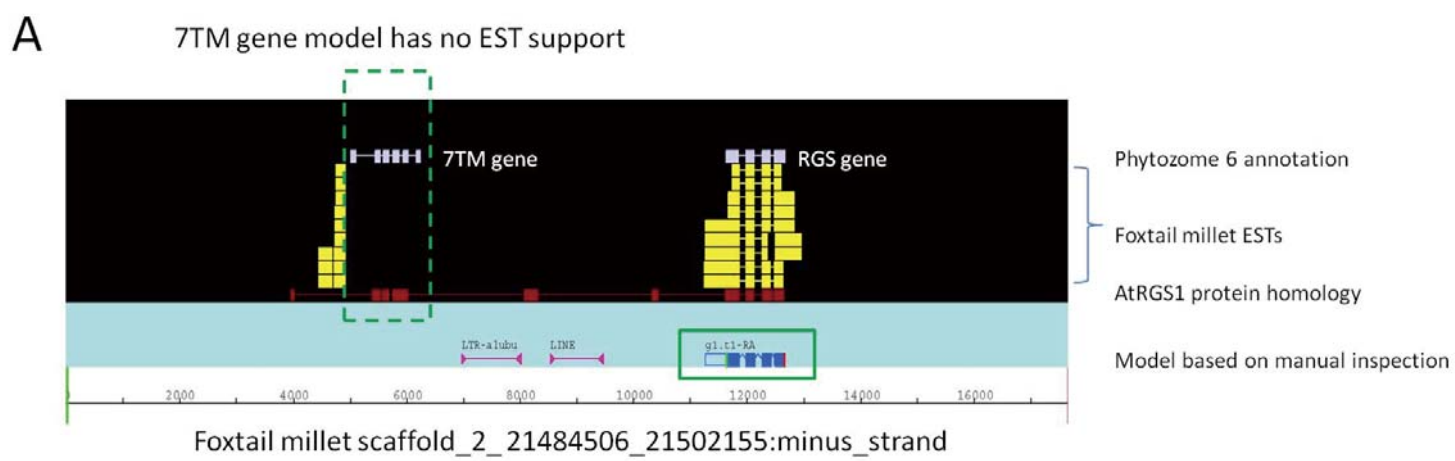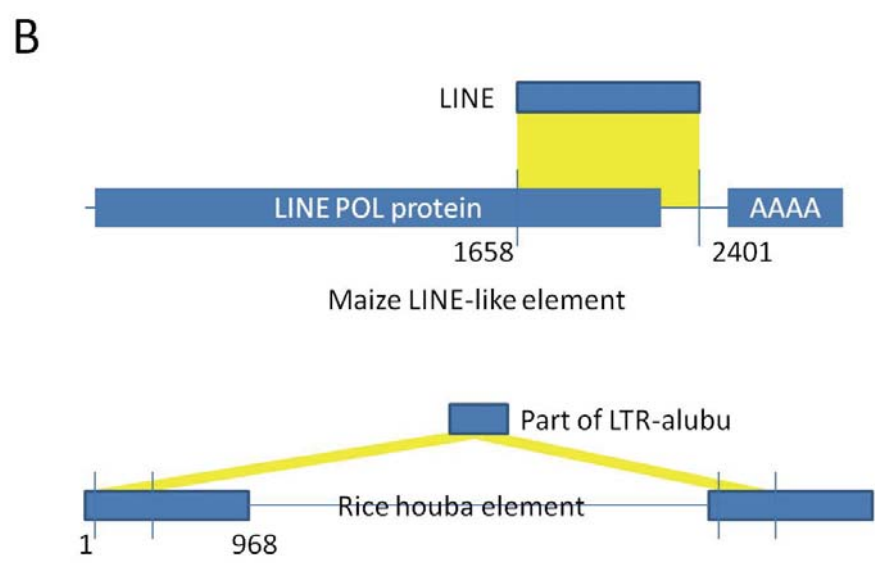

Supplement: Figure S6 — RGS locus in S. italic (foxtail millet). (A) Annotation details of S. italica RGS gene. The final model is within the green box. This model is consistent with RGS gene annotation in Phytozome Setaria versions 6 and 7 (light blue boxes). The dashed line green box shows the position of the 7TM region, which is annotated as a gene in Phytozome v6, but not in Phytozome v7. This region does not have EST support. Two TEs, denoted as LTR-alubu and LINE, are detected between the S. italica RGS gene and the apparently pseudogenized 7TM domain. (B) Scope of the two TEs when using their closest known intact elements as references. (PDF) [file pgen.1002756.s006.pdf]
