# Supplementary material for: G Protein Activation without a GEF in the Plant Kingdom
Source: PLoS Genet. 2012 Jun 28;8(6):e1002756. doi: 10.1371/journal.pgen.1002756 (PMC3386157; doi:10.1371/journal.pgen.1002756)
Supplement: Table S1 — G-protein components in the land plant kingdom. Homologous genes of A. thaliana AtGPA1, AGB1, and AtRGS1 were assembled from plant genome database through Phytozome v7.0 (released on Apr/8/2011; www.phytozome.net), nucleotide or EST database registered in NCBI (www.ncbi.nlm.nih.gov), and EST database of M. polymorpha (http://Marchantia.pmb.lif.kyoto-u.ac.jp). (1) All RGS genes were predicted to have 7- or 5-transmembrane domain, except non-transmembrane S. italica and P. dactylifera RGS. (2) Sequences of G.max G protein components were corrected according to previous research [13], because sequences registered in the soybean genome assembly (www.plantgdb.org/GmGDB/, Soybean Transcript (GenBank 170)) contain some deletions. (3) A Gγ homologous sequence is found highly in the S. moellendorffii genome (scaffold_123: 288795–289362 base) in JGI genome database, although the sequence has not assembled as Gγ gene. (4) A P. patens gene (XP_001772174.1) is incorrectly annotated as Gα in NCBI database. It is highly homologous to Arabidopsis extra-large GTP-binding protein (XP_002890957.1). (5) A P. patens Gγ gene is found in the EST and genome database, although it has not been assembled as a gene. (PDF) [file pgen.1002756.s007.pdf]

	Gα		Gβ		Gγ		RGS[1]		Data source
Arabidopsis thaliana	GPA1	AT2G26300.1	AGB1	AT4G34460.1	AGG1<br>AGG2<br>AGG3	AT3G63420.1<br>AT3G22942.1<br>AT5G20635.1	RGS1	AT3G26090.1	TAIR release 10 acquired from TAIR
Arabidopsis lyrata		481489		491213		873820<br>907928<br>862485		484372	JGI release v1.0
Glycine max	GmGα1<br>GmGα2<br>GmGα3<br>GmGα4	Glyma04g05960.1<br>Glyma14g11140.1<br>Glyma17g34450.1<br>Glyma06g05960.1	GmGβ1<br>GmGβ2<br>GmGβ3<br>GmGβ4	Glyma04g01460.1<br>Glyma06g01510.1<br>Glyma11g12600.1<br>Glyma12g04810.1		Glyma02g16190.1<br>Glyma07g04510.1<br>Glyma10g03610.1<br>Glyma14g17060.1<br>Glyma15g19630.1<br>Glyma17g05640.1<br>Glyma17g29590.1<br>Glyma11g18050.1	RGS1<br>RGS2	Glyma11g37540.1<br>Glyma18g01490.1	The soybean genome assembly(www.plantgdb.org/GmGDB/).<br>See footnote[2] for more information.
Populus trichocarpa		POPTR_0018s08460.1<br>POPTR_0006s23610.1		POPTR_0004s16110.1<br>POPTR_0009s11820.1		POPTR_0005s20180.1<br>POPTR_0005s23760.1<br>POPTR_0006s14330.1<br>POPTR_0018s07510.1<br>POPTR_0015s13280.1<br>POPTR_0002s08200.1<br>POPTR_0002s04770.1		POPTR_0008s18180.1	JGI assembly release v2.0, annotation v2.2
Vitis vinifera		GSVIVT01035483001		GSVIVT01031958001		GSVIVT01015067001<br>GSVIVT01018076001<br>GSVIVT01035600		GSVIVT01010292001	March 2010 12X assembly and annotation from Genoscope

						001			
Cucumis sativus		Cucsa.271110.1		Cucsa.240500.1		Cucsa.016430.1<br>Cucsa.117350.1<br>Cucsa.141810.1<br>Cucsa.310840.1<br>Cucsa.386730.1		Cucsa.075330.1	Roche 454-XLR assembly and JGI v1.0 annotation
Medicago truncatula		Medtr1g019860.1		Medtr3g159480.1		Medtr1g083530.1<br>Medtr2g049340.1<br>Medtr7g034920.1<br>Medtr8g132500.1		Medtr3g102890.1	Release Mt3.0 from the Medicago Genome Sequence Consortium
Manihot esculenta		cassava4.1_009289m<br>cassava4.1_033716m		cassava4.1_009775m<br>cassava4.1_009786m		cassava4.1_024861m<br>cassava4.1_028296m<br>cassava4.1_023456m		cassava4.1_006961m	Assembly version 4, JGI annotation v4.1
Ricinus comunis		29739.m003634		30190.m010890		29648.m002019<br>29739.m003749<br>29844.m003197<br>29883.m001977		29929.m004761	TIGR release 0.1
Mimlus guttatus		mgv1a007908m		mgv1a008244m		mgv1a017061m<br>mgv1a013695m<br>mgv1a016439m		mgv1a005744m	JGI 7x assembly release v1.0 of strain IM62, annotation v1.0
Eucalyptus grandis		Egrandis_v1_0.043077m		Eucgr.I01264.1		Eucgr.G01916.1<br>Eucgr.B01476.1<br>Eucgr.C03259.1<br>Eucgr.H03396.1		Eucgr.H04699.1	JGI v1.0 assembly and annotation
Citrus sinensis		orange1.1g016288m		orange1.1g016458m		orange1.1g033950m<br>orange1.1g034008m<br>orange1.1g037836m<br>orange1.1g0386		orange1.1g018201m	JGI v1.1 annotation on v1 assembly

						68m			
Citrus clementina		clementine0.9_012405m		clementine0.9_012998m		clementine0.9_026147m<br>clementine0.9_023217m<br>clementine0.9_026133m		clementine0.9_009530m	JGI v0.9 assembly and annotation
Prunus persica		ppa006908m		ppa007241m		ppa013611m<br>ppa013749m<br>ppa021140m		ppa005608m	JGI release v1.0
Oryza sativa	RGA1	LOC_Os05g26890.1	RGB1	LOC_Os03g46650.1	RGG2	LOC_Os02g04520.1<br>LOC_Os09g26999.1		Not found	MSU Release 6.0 of the Rice Genome Annotation
Brachypodium distachyon		Bradi2g60350.1		Bradi1g12820.1		Bradi1g14140.1<br>Bradi3g03350.1<br>Bradi4g31420.1		Not found	JGI 8x assembly release v1.0 of strain Bd21 with JGI/MIPS PASA annotation
Sorghum bicolor		Sb01g045320.1		Sb01g012370.1		Sb01g014060.1<br>Sb01g032830.1<br>Sb02g025860.1<br>Sb07g022330.1		Not found	Sbi1.4 models from MIPS/PASA on v1.0 assembly
Zea mays		GRMZM2G064732_T01		GRMZM2G045314_T03		GRMZM2G172320_T01<br>GRMZM2G001660_T01<br>GRMZM2G139878_T01<br>GRMZM2G028726_T02<br>GRMZM2G015578_T03		Not found	Unfiltered protein coding models from Maizesequence.org release 5a.59
Setaria italica		Si022288m		Si036161m		Si039839m<br>Si019618m<br>Si033308m		Si031119m	JGI 8.3X chromosome-scale assembly release 2.0, annotation version 2.1
Phoenix dactylifera		PDK_30s918561g015		PDK_30s660001L033		Not analyzed		PDK_30s709111L00	New Draft Sequence Version 3

Triticum aestivum	TaGA1<br>TaGA2<br>TaGA3	AB090158.2<br>AB090159.1<br>HQ020506.1	TaGB1	AB090160.1		Not analyzed		Not found	Nucleotide database in NCBI
Hordeum vulgare		AF267485.2		AK251844		Not analyzed		Not found	Nucleotide database in NCBI
Pinus taeda	PtGα1	Cloned in this study		DR692385.1/CX651314.1/DR047972.1		CX650880<br>DT638145<br>R093761.1	PtRGS1	Cloned in this study	EST database in NCBI
Picea glauca		Cloned in this study		EX353861.1/EX406541.1		EX423497<br>EX374535.1<br>EX380234.1		BT111406	EST database in NCBI
Selaginella moellendorffii	GPA-1	XM_002960950.1	AGB1-1	XP_002964323.1		See footnote[3]		XM_002984541.1	Nucleotide database in NCBI
Marchantia polymorpha	MpGα1	isotig19630		isotig30299		isotig30025		Not found	EST database (See footnote).
Physcomitrella patens		Not found[4]		Pp1s7_401V6.2<br>Pp1s28_162V6.1		Pp1s22_182V6.1<br>CAYZ19681[5]		Not found	JGI assembly release v1.1 and COSMOSS annotation v1.6 or<br>EST database in NCBI
Volvox carteri		Not found		Not found		Not found		Not found	GenBank annotations based on JGI v1.0
Chlamydomonas reinhardtii		Not found		Not found		Not found		Not found	Augustus update 10.2 (u10.2) annotation of JGI assembly v4

Table S1. G-protein components in the land plant kingdom

Homologous genes of *A. thaliana* AtGPA1, AGB1, and AtRGS1 were assembled from plant genome database through Phytozome v7.0 (released on Apr/8/2011; www.phytozome.net), nucleotide or EST database registered in NCBI (www.ncbi.nlm.nih.gov), and EST database of *M. polymorpha* (http://*Marchantia*.pmb.lif.kyoto-u.ac.jp). (1) All RGS genes were predicted to have 7- or 5-transmembrane domain, except non-transmembrane *S. italica* and *P. dactylifera* RGS. (2) Sequences of *G.max* G protein components were corrected according to previous research [13], because sequences registered in the soybean genome assembly (www.plantgdb.org/GmGDB/, Soybean Transcript (GenBank 170)) contain some deletions. (3) A Gγ homologous sequence is found highly in the *S. moellendorffii* genome (scaffold_123: 288795-289362 base) in JGI genome database, although the sequence has not assembled as Gγ gene. (4) A *P. patens* gene (XP_001772174.1) is incorrectly annotated as Gα in NCBI database. It is highly homologous to Arabidopsis extra-large GTP-binding protein (XP_002890957.1). (5) A *P. patens* Gγ gene is found in the EST and genome database, although it has not been assembled as a gene.
