# Supplementary material for: G Protein Activation without a GEF in the Plant Kingdom
Source: PLoS Genet. 2012 Jun 28;8(6):e1002756. doi: 10.1371/journal.pgen.1002756 (PMC3386157; doi:10.1371/journal.pgen.1002756)
Supplement: Table S2 — BLAST search of S.italica 7TM-RGS gene. Genomic sequence of S. italica (Segment ID: 13, Bases: 1356001–1363646) was used as query for BLASTx against A. thaliana non-redundant protein sequences registered in NCBI. Results shown here are homologous sequences to AtRGS1 to fill a gap between SiPROV019851m and SiPROV032159m, which were similar to a part of 7TM-RGS gene. (PDF) [file pgen.1002756.s008.pdf]

SIbac13 1360549 1360367	VFHWNKPLNHRCHMQAQWVIPVMCIHGFYIAGLIVITLSIRHIEFRFSEFKDLLQAIIVST
	+ H KPLN +CHM QW PV +H Y+ LI T ++RH+EFRF E +DL + I+VS
AtRGS1 mRNA 424	IIHATKPLNDKCHMGLQWTFPVAGLHALYVLALIAFTRAVRHVEFRFDELRDLWKGILVSA 606
SIbac13 1358329	TSTGFWIVAYVLNEIHEDIAWIQVFSRFSLLV 1358234
	TS W+ A+VLNEIHE+I+W+QV SRF LLV
AtRGS1 mRNA 607	TSIVIWVTAFVLNEIHEEISWLQVASRFVLLV 702
SIbac131357038	ILVLLFFSMSISQPLHSQISLGKQESTAFMTMGEALGITDRG 1356913
	ILV++FFS+S +QPL SQISL K+++ F MG+ALGI D G
AtRGS1 mRNA 712	ILVVVFFSISSNQPLLSQISLKKRQNFEFQRMGQALGIPDSG 837

Table S2. BLAST search of *S.italica* 7TM-RGS gene

Genomic sequence of *S. italica* (Segment ID: 13, Bases: 1356001 - 1363646) was used as query for BLASTx against *A. thaliana* non-redundant protein sequences registered in NCBI. Results shown here are homologous sequences to AtRGS1 to fill a gap between SiPROV019851m and SiPROV032159m, which were similar to a part of 7TM-RGS gene.
