# Supplementary material for: G Protein Activation without a GEF in the Plant Kingdom
Source: PLoS Genet. 2012 Jun 28;8(6):e1002756. doi: 10.1371/journal.pgen.1002756 (PMC3386157; doi:10.1371/journal.pgen.1002756)
Supplement: Table S3 — dn/ds values at 7TM and RGS domains. dN, the number of non-synonymous mutations per sites. dS, the number of synonymous mutations per sites. (PDF) [file pgen.1002756.s009.pdf]

Domain	Species compared		dn	ds	dn/ds
7TM	*A. thaliana*	*S. italica*	0.455	1.767	0.257
	A. thaliana	*P. dactylifera*	0.306	1.4861	0.206
	S. italica	*P. dactylifera*	0.2596	1.5451	0.168
RGS	*A. thaliana*	*S. italica*	0.2126	3.2996	0.064
	A. thaliana	*P. dactylifera*	0.168	3.3134	0.051
	S. italica	*P. dactylifera*	0.1424	1.0539	0.135

Table S3. dn/ds values at 7TM and RGS domains. dN, the number of non-synonymous mutations per sites. dS, the number of synonymous mutations per sites.
